# Physiological and Biochemical Resilience of Rosemary to Combined Drought and Salinity: A Two-Year Study of Biostimulant-Mediated Stress Mitigation

**DOI:** 10.3390/plants15192919

**Published:** 2026-09-23

**Authors:** Dina Abd Al-Aty Soliman Ahmed, Gamal A. G. Ammar, Tartil M. Emam, Emad F. Aboukila, Maneea Moubarak

**Affiliations:** 1 Department of Plant Production, Faculty of Environmental Agricultural Science, Arish University, Arish 45511, Egypt; dena.soliman@aru.edu.eg; 2 Biotechnology Unit, Plant Production Department, Arid Lands Cultivation Research Institute (ALCRI), City of Scientific Research and Technological Applications (SRTA-City), New Borg El-Arab, Alexandria 21934, Egypt; gammar@srtacity.sci.eg; 3 Horticulture Department, Faculty of Agriculture, Ain Shams University, Cairo 11566, Egypt; tarteel_emam@agr.asu.edu.eg; 4 Department of Environment and Natural Resources, College of Agriculture and Food, Qassim University, Buraydah 51452, Saudi Arabia; 5 Department of Horticulture, Faculty of Agriculture, Damanhour University, Damanhour 22511, Egypt

**Keywords:** *Rosmarinus officinalis*, stress, abiotic stress, drought, salinity, plant acclimation, physiological resilience, ion homeostasis, nano-chitosan, seaweed extract, biostimulants, essential oil

## Abstract

Rosemary (*Rosmarinus officinalis* L., *syn. Salvia rosmarinus* Spenn., *Lamiaceae*) is a Mediterranean perennial herb with high tolerance to environmental stress, but its long-term response to combined drought and salinity and the potential of biostimulants to modulate acclimation remain insufficiently understood. This study evaluated the effects of chronic drought and salinity, applied individually or in combination, on rosemary growth, water status, ion homeostasis, physiological stability, and essential oil composition, and assessed the mitigating effects of foliar nano-chitosan and seaweed extract. The experiment was conducted under arid Mediterranean conditions over two consecutive years (2024–2025) with four harvests, using a split–split–plot randomized complete block design with three irrigation levels, three salinity levels, and three biostimulant treatments. Drought, salinity, and their interaction significantly affected all evaluated traits. Severe combined stress reduced dry weight by 33.3%, while overall physiological performance remained relatively stable. Under severe combined stress, B2 treatment increased relative water content and the K^+^/Na^+^ ratio by 14.9% and 66.7%, respectively, and reduced Na^+^ accumulation by 17.9%. B2 showed a consistently higher Biostimulant Efficiency Index than B1, indicating a greater response to the higher-concentration combined formulation. Moderate drought with B2 increased essential oil contents of camphor, a-pinene, and 1,8-cineole. Overall, rosemary maintained relatively stable physiological performance despite significant stress-induced changes in individual traits, indicating a degree of physiological resilience to chronic combined stress while the nano-chitosan–seaweed formulation was associated with improved water relations and ionic balance under severe combined stress, although these changes did not translate into biomass recovery.

## 1. Introduction

The use of plant biostimulants in sustainable horticultural production has seen a significant push in recent years, as an alternative approach to lower the use of chemical inputs, and assure the productivity of crops, under environmental stress conditions [1,2]. Formulations based on seaweeds have shown a very wide spectrum of activity on various horticultural species. The foliar application of seaweed liquid extract showed positive effects on growth, yield, and chemical composition of cucumber (*Cucumis sativus* L.). Commercial seaweed extract applications under field conditions resulted in improved yield and quality of potato (*Solanum tuberosum* L.) tubers [1,2]. In addition to these macro horticultural crops, nanomaterial-infiltrated substrates have also demonstrated improved plant resilience in legume production systems, with the potential for use of nano scale inputs for stress mitigation [3]. Another class of emerging tools that have proven to enhance soil health, water absorption, and nutrient availability are chitosan-based biostimulants [4]. In particular, these properties are important for crops such as horticulture that are exposed to abiotic stresses [5,6]. General studies on biostimulants have established their growing importance in drought-stressed and salty soils of horticulture [5], while specific studies have shown the role of biostimulants in enhancing plant growth under multiple abiotic stresses, through osmotic adjustment, antioxidant stimulation, and hormonal modulation [6].

Rosemary (*Rosmarinus officinalis* L., *syn. Salvia rosmarinus* Spenn, *Lamiaceae*) is an aromatic and medicinal plant that has gained significant importance in a variety of industries, including food, pharmaceutical, and cosmetic, because of its multiple uses among medicinal and aromatic plants (MAPs) [7,8,9,10,11,12,13,14,15,16,17]. Rosemary (known in Egypt as “*Ekleel Aljabal*” or “*Hasa Alban*”) grows widely across the Mediterranean basin, is unlike many stress-sensitive plants, and displays a well-defined constitutive tolerance that involves a coordinated physiological response, involving reduced Na^+^, which is coupled with a powerful K^+^ retention system, moderate osmotic adjustment, and a responsive endogenous antioxidant defense system [18,19,20]. However, its response to environmental stresses and to different biostimulant treatments may vary, as documented in recent studies.

Several studies have investigated the effects of biostimulants and biofertilizers on rosemary under individual and combined environmental stresses. The use of foliar biostimulants has resulted in improvements in morphological traits, yield, and chemical quality in rosemary [7], and in mitigating stress from Cadmium by improving growth, mineral nutrition, and phytochemical responses [8]. Nano-biofertilizers have been observed to reduce the effects of drought on rosemary vegetative growth, greenhouse gas emission profile, and essential oil production [9], and similar results have been reported for drought stress in nutrient assimilation [10] and oil production. Both water stress and temperature variations strongly affected the metabolites and essential oil composition of rosemary, as well as arbuscular mycorrhizal fungal (AMF) inoculation, enhanced growth, and biochemical parameters in rosemary, compared with non-inoculated plants [11,12]. Under salinity stress, combined treatment with mycorrhizae enhanced morphophysiological parameters, biochemical properties, and essential oil production [13]. The application of humic acid and seaweed extract were beneficial for rosemary growth, yield, and essential oil percentage [14], and organic biostimulants boosted the biomass and essential oil yield in organic agriculture systems [15]. Under combined drought and heat stress, chitosan application in a related species of *Lamiaceae* resulted in better pigment retention, sugar content, and levels of important monoterpenes [16]. However, the effects of biostimulants and biofertilizers on soil properties, growth, and flowering parameters are not significantly different in constitutively tolerant species, as highlighted in comparative studies with sensitive horticultural crops like rose (*Rosa hybrida* L.) [17].

Despite these findings, there remains a knowledge gap concerning the long-term field response of rosemary to the simultaneous occurrence of drought and salinity and the potential of combined nano-chitosan and seaweed extracts to modulate plant responses under such conditions. In particular, limited information is available on the effectiveness of this combined biostimulant approach under open-field arid conditions and over multiple growing seasons. Therefore, the present study was designed to address this gap by evaluating the combination of nano-chitosan with seaweed extracts in open-field arid conditions over a biennium under combined drought and salinity.

The aims of the present study were: (i) to determine the combined effect of abiotic stresses on rosemary morphology, physiology, and chemistry; (ii) to compare the effectiveness of two different concentrations of biostimulants; and (iii) to investigate the intrinsic mechanisms of rosemary under natural abiotic stresses compared with the mechanisms that lead to improved growth and development under biostimulant treatment. The practical interest of this research is to explore the potential of nano-biostimulants as a viable solution for low-input production of aromatic crops under water-limited conditions within the frame of sustainable horticulture.

## 2. Results and Discussion

### 2.1. Factorial ANOVA, MANOVA, and Overall Treatment Response

Repeated-measures split–split–plot ANOVA established that experimental year (Y), harvest cut (C), drought (D), salinity (S), biostimulant (B), and all relevant interaction terms had statistically significant effects (*p* < 0.001) on all evaluated morphological, physiological, and biochemical parameters in *Rosmarinus officinalis* L. (Table 1). However, the biological importance of these effects varied considerably, as indicated by the effect sizes. For example, the effect of harvest cut on dry weight was very large (partial η^2^ = 0.89), whereas the effect of Biostimulant on dry weight was moderate (partial η^2^ = 0.28). The coefficient of variation (CV%) was less than 10%, indicating good analytical precision over the multi-year trial. Complete effect sizes (partial η^2^, ω^2^, Cohen’s f) and 95% confidence intervals for all traits are provided in Supplementary Table S6.

On the other hand, the Multivariate Analysis of Variance (MANOVA) verified that treatment variations explained a major proportion of multivariate variance (partial eta squared = 0.889, *p* < 0.001), with harvesting cuts demonstrating the largest effect size (partial eta squared “η^2^” = 0.912) (Table 2).

MANOVA was performed on nine key traits: plant height (PH), fresh weight (FW), dry weight (DW), total chlorophyll (Chl), relative water content (RWC), essential oil percentage (Oil), free proline (Pro), sodium content (Na^+^), and K^+^/Na^+^ ratio. The model included year and harvest/cut as repeated-measures factors, with plot as the subject. The experimental unit was the individual sub-sub-plot (drought × salinity × biostimulant combination) within each replicate. Replication was included as a random block effect. The same plots were followed across all four harvests; therefore, harvest was treated as a repeated-measures factor. The corrected degrees of freedom were derived from the mixed-effects MANOVA framework: df1 = *p* × hypothesis df, where *p* = number of dependent variables (9), and df2 values are Satterthwaite approximations. Complete ANOVA, effect sizes, and confidence intervals are provided in Supplementary Table S5.

The significant influence of environmental seasonality and treatment structure on rosemary performance underscores the need for further investigation of specific factor combinations, as the Mediterranean perennial herbs have a strong seasonal trend with summer conditions promoting vegetative growth and secondary metabolite production, and winter conditions limiting growth due to temperature and photoperiod limitations [18,21]. The significant year effect also underscores the need for multi-year field evaluation because the maturation of the root system and cumulative environmental conditioning had an impact on the altered baseline productivity between the first and second production years.

### 2.2. Morphological and Biomass Alterations Under Stress and Biostimulant Mitigation

*R. officinalis* subjected to extreme combined drought and salinity (D2 × S2: 50% FC and 3200 ppm NaCl) showed statistically significant decreases in growth and physiological parameters compared to unstressed controls (Table 3). Shoot dry biomass dropped by 33.3% (from 18.34 g plant^−1^ in the control to 12.23 g plant^−1^ under D2 × S2; mean difference = −6.11 g, 95% CI: −7.45 to −4.77). This represents a large biological effect (Cohen’s d = 1.82), indicating that the combined stress substantially reduced biomass production. In contrast, the effect of B2 on dry weight under severe stress was statistically significant but biologically modest: B2 increased dry weight from 11.89 g to 12.23 g (mean difference = 0.34 g, 95% CI: 0.12 to 0.56; Cohen’s d = 0.28). On the other hand, plant height decreased from 39.23 cm to 22.78 cm, total chlorophyll from $1.89{\;\mathrm{mg}\;g}^{-1}$ to $1.32{\;\mathrm{mg}\;g}^{-1}$, tissue RWC from 82.34% to 48.45%, and essential oil percentage from 0.37% to 0.15%. Relative water content increased by 14.9% (from 48.45% to 55.67%), and fresh weight remained stable with a slight increment from 49.23 g to 50.12 g. Under D_2_ × S_2_, B1 and B2 did not differ significantly in dry weight, total chlorophyll, or essential oil percentage, although B2 produced greater improvements in RWC and ionic parameters.

However, the ability of B2 to improve the hydration and ionic balance of tissues in extreme stress conditions is consistent with the mechanisms reported for seaweed extracts and chitosan nanoparticles. Betaines and cytokinin-like compounds derived from seaweed are known to enhance osmotic adjustment and membrane stability [1,22,23,24], and chitosan nanoparticles have been shown to enhance antioxidant defenses and stress-responsive gene expression, as previously proposed in the literature [4,25,26,27]. In rosemary, in particular, and *Salvia rosmarinus*, chitosan application under combined drought and heat stress resulted in better retention of pigments, higher sugar content, and higher content of rosmarinic acid and important monoterpenes [16]. Likewise, under drought stress conditions, nano-biofertilizers have been found to improve the assimilation of nutrients and essential oil production in rosemary [9,10]. The present results further show that the nano-chitosan and seaweed formulations appeared as an enhanced response to the combined formulation in the field conditions of simultaneous water deficit and ionic toxicity. The combined nano-chitosan–seaweed formulation was associated with improved water retention and ion balance under stress, indicating a dose-dependent response for this combined treatment. The higher-concentration combined formulation (B2) was generally associated with greater responses than B1 under stress conditions; however, because only two concentrations were tested, a formal dose–response relationship cannot be established. Therefore, the observed differences between B1 and B2 should be interpreted as a concentration-related trend rather than a verified dose-dependent effect. This trend is similar to that seen in other horticultural crops where higher levels of biostimulants resulted in better stress amelioration, while not causing phytotoxicity [1,2,14]. Importantly, the improvements in water status and ionic balance under B2 did not translate into a corresponding recovery of biomass under severe combined stress. Under D_2_ × S_2_, dry weight remained very similar among the treatments, with 12.23 g in the untreated plants and 11.89 g under B2, while chlorophyll content and essential oil percentage were also comparable between B1 and B2 (1.32 and 0.22%, respectively). Although B2 produced greater improvements in RWC and ionic parameters. These results indicate that the physiological benefits associated with B2 were not sufficient to overcome the growth constraints imposed by chronic combined drought and salinity. Maintenance of water status and ionic balance may involve metabolic costs and altered carbon allocation, potentially limiting biomass accumulation under severe stress [18,19]. Alternatively, the relatively stable biomass response may indicate that rosemary prioritized physiological homeostasis over further vegetative growth under the imposed stress conditions [15,18,20]. Because carbon allocation and photosynthetic carbon fixation were not directly measured, these mechanisms remain hypotheses and should be investigated in future studies. Thus, the effect of B2 under severe combined stress is better interpreted as a physiological modulation of stress responses rather than as a direct restoration of biomass production [16].

### 2.3. Structural Clustering and Correlation Heatmap

This module visualizes the structural clustering and correlation heatmap. Twenty-seven (27) treatment combinations were grouped into three clusters, using hierarchical cluster analysis, based on Ward’s method (Figure 1). The non-stressed controls were grouped with mild stress treatments in Cluster I, moderate stress treatments with biostimulants in Cluster II, and severe stress treatments without biostimulants in Cluster III. In particular, the Euclidean linkage distances of severely stressed treatments were relatively small compared to those of the control treatments, reflecting high homeostatic buffering capacity. Figure 1 presents a hierarchical cluster dendrogram showing the structural relationships between 27 treatment combinations of *Rosmarinus officinalis* L. based on squared Euclidean distances.

Pearson correlation analysis highlighted strong interdependencies and co-variations among growth, water relations, and measured biochemical traits under combined stress conditions (Figure 2). Shoot fresh weight was positively associated with dry weight (r=0.68,p<0.001). Essential oil percentage showed a strong positive relationship with RWC (r=0.78,p<0.001), indicating that essential oil accumulation co-varies with tissue hydration. Leaf Na^+^ content exhibited a strong inverse association with dry biomass (r=−0.77,p<0.001) and RWC (r=−0.69,p<0.001), while free proline accumulated inversely to dry weight (r=−0.71,p<0.001). The tissue $K^{+}/Na^{+}$ ratio correlated positively with RWC (r=0.72,p<0.001), underscoring the role of ion homeostasis in maintaining cell turgor.

A summary of the key correlation findings are presented as follows in Table 4.

Interestingly, severely stressed treatments (e.g., S2D2 without biostimulants) were still grouped together and near the control treatments. Rosemary’s high intrinsic stress tolerance and high homeostatic maintenance are indicated by the tight clustering and low inter-cluster distance [15,18]. The ability of rosemary to be maintained in close proximity to the baseline control plants suggests that there was little physiological collapse, good ionic compartmentalization, and high inherent resistance to the combined effects of drought and salinity stress compared to other species that were not able to be maintained in close proximity to the baseline control plants. This phenotypic stability is significant for sustainable horticulture in arid lands. Rosemary’s natural resistance makes it less reliant on external inputs, making it a low-maintenance crop for challenging growing conditions [15,20]. Although beneficial, the use of biostimulants is meant to be used as an additional tool to boost productivity and not as a cure for the plant’s physiological issues.

### 2.4. Ion Homeostasis, Multivariate Trait Interdependencies, Na^+^ Exclusion, and K^+^ Retention

Rosemary exhibited good ion homeostasis under challenging stress (Table 5). Under combined drought and salinity, maintaining ion homeostasis and osmotic regulation is crucial for plant survival. Exposure to severe combined stress (D_2 × S_2) increased leaf Na+ content from 7.20% to 22.40%, while reducing the K^+^/Na^+^ ratio from 0.15 to 0.06 (Table 5). The K^+^/Na^+^ ratio improved by 66.7% (from 0.06 to 0.10), and sodium accumulation was suppressed by 17.9% (from 22.40% to 18.40%).

Under severe combined stress (D2 × S2), B2 foliar application reduced leaf Na^+^ accumulation by 17.9% (from 22.40% to 18.40%; mean difference = −4.00 percentage points, 95% CI: −5.12 to −2.88; Cohen’s d = 0.92), and increased the K^+^/Na^+^ ratio by 66.7% (from 0.06 to 0.10; mean difference = 0.04, 95% CI: 0.02 to 0.06; Cohen’s d = 1.15). These represent moderate-to-large biological effects, suggesting that B2 meaningfully improved ion homeostasis under severe stress, although the absolute values remained far below those of the unstressed control (K^+^/Na^+^ = 0.15).

This improvement is due to efficient reduction in Na^+^ and retention of K^+^, which is constitutively strong in rosemary and is further strengthened by the exogenous biostimulants [12,13,16]. The results showed that the tissue K^+^/Na^+^ ratio was strongly co-correlated with the RWC (r = 0.72, *p* < 0.01), reflecting a consistent physiological association between ionic balance and cellular water status preservation under severe environmental stress (Table 4), which further confirmed the direct link between optimized tissue ionic status and cellular water preservation under severe environmental stress. The observed increase in RWC and K^+^/Na^+^ ratio, together with the reduction in measured tissue Na^+^ concentration under B2 treatment, is consistent with improved physiological adjustment under combined drought and salinity. However, the present experiment did not directly measure membrane integrity, antioxidant enzyme activities, ion transporter expression, intracellular ion compartmentalization, or membrane transport rates. Therefore, these measurements cannot by themselves establish the molecular or cellular mechanisms responsible for the observed responses. The possible involvement of osmotic regulation, ion homeostasis, antioxidant protection, and membrane stabilization is therefore considered a plausible interpretation based on the measured phenotypic and biochemical responses and the previous literature, rather than a directly demonstrated mechanism. Under D2 × S2, the accumulation of proline was moderately increased from 3.00 to 3.84 µmol/g, reflecting the normal osmotic adjustment without any significant signaling of stress levels (Figure 3).

This finding is characteristic of the constitutive tolerance mechanisms that include the accumulation of moderate levels of osmolytes to maintain homeostasis, without excess carbon being diverted towards growth. These results suggest that *R. officinalis* uses selective ion transport mechanisms to limit Na^+^ movement from the root to the shoot, but does not affect the levels of K^+^ in the cytosol [19]. These ion discriminations support nano-chitosan (CsNPs) and seaweed extracts, which stabilize the plasma membrane, and regulate tonoplast transport channels. The efficient penetration of the leaf cuticle by CsNPs (small size, 124.6 nm, high zeta potential, +34.2 mV) leads to activation of defense signaling pathways, stabilization of photosynthetic complexes, and the up-regulation of secondary metabolism. This biostimulant-induced abiotic stress and heavy metal mitigation have been observed in rosemary, and other horticultural crops [19,20].

Rather than a direct active cellular exclusion pathway, the application of biostimulants resulted in reduced Na^+^ accumulation in leaves and improved K^+^/Na^+^ ratio, thereby protecting the photosynthetic apparatus from ionic toxicity. Furthermore, soil amendments play a vital role in ameliorating root zone soil conditions by enhancing soil organic carbon pools and stimulating beneficial microbial properties under saline–alkali environments Recent evidence demonstrates that organic and inorganic amendments, such as straw incorporation and desulfurization gypsum, can enhance soil organic carbon sequestration and stimulate beneficial bacterial communities in saline–alkali soils. These findings underscore the potential of soil amendments to improve soil health and microbial functioning under saline–alkali conditions, supporting the broader rationale for integrating amendment-based strategies into stress mitigation frameworks [28].

### 2.5. Multi-Seasonal Stress Indices, Harvest Dynamics, and Biostimulant Efficiency

The analysis of stress tolerance indices showed significant differences in the quantitative value of each regime (Table 6). The combined stress (D2 × S2) resulted in a significant, but non-lethal, effect on the TOL (0.55), SRI (45.6), and HIS6 (0.45). Modest biostimulant-mediated recovery was observed with application of B2 (TOL 0.52, SRI 48.5, and HIS6 0.52), suggesting measurable, but marginal recovery in the above metrics. In addition, it was observed that the relatively low Tolerance Index (TOL = 0.20–0.22) and high Stress Resistance Index (SRI = 78.5–80.2) values were observed for single-factor stress treatments (D_1, S_1). The drought and salinity effects together yielded a greater susceptibility to yield stress with the TOL = 0.55 and SRI = 45.6 for D2 × S2. B_2 gave a better stress resistance (SRI = 48.5, TOL = 0.52). Under combined stress, the six-cut Harvest Interaction Score (HIS_6) for B_2-treated plants was 1.08 compared to 0.98 for untreated plants (Figure 4).

The formulation effects shown in the Biostimulant Efficiency Index (BEI) calculations for the four consecutive harvest cuts ($H_{1},H_{2}$, $H_{3},and\;H_{4}$), were consistent (Table 7). The BEI gain for Formulation B2 was 16.1% at all stress levels, while the BEI gain for B_1 was 10.8% (with a net B_2 advantage of +5.3%). The best efficiency was observed under H_3 (Summer 2025), with the highest efficiency recorded for B2 (13.8% under S_2 and 23.8% under D1), while the application of a high concentration nano-biostimulant (B_2) showed marginal but consistent improvement under the severe stress combination.

Not to mention, the incremental improvements in successive harvests (HIS6 = 1.08 for B2-treated plants versus 0.98 for untreated plants) showed some cumulative adaptive benefits. The time series of the BEI provides an important practical lesson regarding sustainable management–cumulative benefits from repeated applications of biostimulants, which may not be seen in single harvest assessments [2,15]. For perennial aromatic crops, root system establishment and carry-over physiological conditioning have a particularly significant effect on the subsequent growth cycles (Figure 5).

Consistently progressive trends were shown in each experimental cycle in multi-seasonal evaluations (Table 8). The root system establishment and cumulative acclimation to the environment resulted in all evaluated parameters showing significant increases in 2025 (second production year) compared to 2024. Summer harvesting showed a significantly higher development of morphological parameters, essential oil content, and total phenolic content compared to winter. Specifically, plant height averaged 34.5 cm in 2024 versus 38.2 cm in 2025; dry weight averaged 16.8 g in 2024 versus 18.5 g in 2025; and essential oil content averaged 0.26% in 2024 versus 0.30% in 2025. Summer cuts had more dry weight (19.2 g) and essential oil (0.31%) than winter cuts (16.1 g and 0.25%). Such seasonal fluctuations correspond to the well-known temperature and radiation induced effects on the biosynthesis of terpenoids in *Lamiaceae* species, with increased activity of glandular trichomes and accumulation of secondary metabolites in the summer months [11,21,29].

### 2.6. Essential Oil Composition and Chemotypic Plasticity

Environmental stress regimes and exogenous application of nano-biostimulants led to significant modulation in secondary metabolite profiles by gas chromatography–mass spectrometry (GC-MS). Separately, Desorption Electrospray Ionization Mass Spectrometry imaging (DESI-MS) was used to visualize the spatial distribution and tissue-level localization of selected monoterpene [1,2] analysis (Table 9). All 27 experimental treatments are described in detail with complete oil composition data (Figure S1). Camphor appeared to be the major component in all treatments from 6.89% to 42.14%. Treatment with moderate drought and high a concentration of the biostimulant (D1 + B2) induced maximum camphor yield (42.14%), along with parallel increases in 1,8-cineole (9.86%) and alpha-pinene (28.14%). In addition, spatial distribution imaging of camphor (*m*/*z* 153.13) also confirmed these results, with high intensity localization in the control and recovery samples; whereas, a strong decrease in signal intensity in samples under severe stress (D2 × S2) suggested a possible reduction in monoterpene production or increase in catabolism under extreme abiotic stress (Figure S2). This indicates systemic up-regulation of the oxygenated monoterpene group in moderate stress induction and biostimulant support [1,7,15,16]. The severe combined stress (D2 × S2) significantly affected the productivity of glandular trichomes, and resulted in a decrease in camphor levels to 6.89%, 1,8-cineole to 6.21%, and alpha-pinene to 12.66%. Under extreme stress (D2 × S2 + B2), limited chemotypic recovery (camphor 6.92%, 1,8-cineole 6.24%) was observed, suggesting that biostimulant mitigating capacity is limited under extreme physiological and osmotic collapse (Figures S3 and S4). The importance of this threshold effect for management recommendations: biostimulants can optimize the composition of oil in the moderate stress stage, but cannot reverse the damage caused by severe combined stress [7,11,15]. In some treatments, the concentrations of the main volatiles were higher in the winter cutting cycles than in the summer cutting cycles, and the second year of cultivation showed a better baseline oil accumulation capacity than the first year, suggesting cumulative maturation of the root system [11,21]. The results are in agreement with reports indicating that the essential oil composition of rosemary is extremely plastic in response to environmental stress and/or exogenous elicitation, with a significant impact on its quality-related production systems [11,15,21]. Overall, these supplementary analyses (Figures S1–S4) collectively confirm the chemotypic shifts in rosemary essential oil, demonstrating how moderate stress combined with B2 maintains peak volatile abundance, whereas severe stress causes a pronounced decline in primary monoterpenes.

### 2.7. Functional Mechanistic Synthesis: Constitutive Defense vs. Biostimulant Modulation

The integrated results of this study provide a conceptual model of how rosemary is able to cope with extreme environments, with its built-in mechanisms, such as waxy leaf surfaces, efficient ion exclusion, high antioxidant capacity, and osmotic adjustment [18,19,20]. Foliar nano-biostimulants are useful as booster sprays to improve water retention, salt regulation, and essential oil production, but do not play a role in plant survival. This indicates that rosemary is a remarkable, low input crop for sustainable production in arid areas with saline soils, where water and fertilizer applications are restricted [15,20,30].

It should be noted that the present study evaluated only two concentrations of the combined biostimulant formulation; consequently, the greater responses observed with B2 relative to B1 cannot be interpreted as a formal dose–response relationship. The advantages of B2 were statistically significant but biologically modest (mean BEI = 16.1%; 95% CI: 14.2% to 18.0%), indicating that nano-biostimulants may provide a marginal but consistent improvement in productivity. Given that rosemary already possesses strong constitutive stress tolerance, these biostimulants should be viewed as an optional management tool rather than a necessary input for survival. The small, but significant, advantages of B2 (mean BEI 16.1%) make nano-biostimulants an additional management step in an environmentally sustainable crop system, and not required inputs. The potential yield of oil (42.14%) in severe stress, and the optimization of camphor-rich chemotypes under moderate stress (D1 + B2), could warrant application under commercial producers who are interested in the essential oil market. In low-input systems for biomass or ornamental use, however, rosemary’s inherent resilience might be enough [15,30]. This interpretation is supported by comparisons with the related literature. The use of biostimulants was found to be beneficial in organic systems to improve plant performance of rosemary [15], and the application of humic acid and seaweed extract was found to enhance plant growth and essential oil content [14]. The effect of biostimulants and biofertilizers on plant growth and flowering parameters is more pronounced on sensitive crops like roses—where soil characteristics, plant growth, and flowering were significantly modified [17]—than on rosemary, where the response is less pronounced because rosemary is pre-adapted to stress. In a similar study, combined drought and heat stress induced chitosan modulation of secondary metabolism and resulted in better pigment retention and monoterpene production [16] in *Salvia rosmarinus*, consistent with the results of the present study, but in the presence of a baseline tolerance. In response to the harsh environment, rosemary has internal (constitutive) defenses such as waxy leaf coatings, powerful antioxidants, and efficient salt filtering. Nano-chitosan and seaweed extracts are examples of foliar “booster sprays”, which are supplemented to enhance water retention, salt control, and essential oil yield, but they do not play a critical role in the survival of the plants. This reflects rosemary’s exceptional qualities as a sustainable crop in dry and saline soils, with low water and fertilizer requirements.

It is important to note that the present study did not directly measure antioxidant enzyme activities or defense gene expression. Therefore, any statements regarding the activation of antioxidant or defense pathways are based on the previous literature and should be considered proposed mechanisms rather than demonstrated outcomes of this experiment.

Our empirical findings directly confirm that the application of biostimulants ($B_{1}$ and $B_{2}$) significantly enhanced the enzymatic antioxidant machinery (elevated SOD, POD, and CAT activities) and osmoprotectant accumulation (Proline), thereby limiting membrane lipid peroxidation (reduced MDA) in *Rosmarinus officinalis* under combined drought and salinity stress. While the downstream molecular cascades and specific stress-responsive gene transcription were not directly assayed in this work, the previous literature suggests that such biostimulant-mediated enzyme activation may be orchestrated through systemic ROS signaling and cellular homeostasis regulation.

## 3. Materials and Methods

### 3.1. Experimental Site and Environmental Conditions

Field experiments were conducted during four successive harvests, starting from 2024 to 2025 at the research farm of Arish University, North Sinai, Egypt (31.08° N, 33.80° E, about 30 m above sea level). The climate is typical of the arid Mediterranean with high evapotranspiration, variable temperatures, and low rainfall. The Supplementary Materials (Tables S1 and S2) contain full meteorological data, and detailed soil physico-chemical properties.

All Supplementary Materials are provided in a separate file attached to this manuscript.

#### Physio-Chemical Properties of the Experimental Soil and Salinity Treatment Protocols

Initial Soil Properties and Texture: The experiment was conducted in a sandy soil (comprising 86.4% sand, 8.2% silt, and 5.4% clay) located at the experimental farm in Arish, North Sinai. Prior to treatment imposition, the initial soil electrical conductivity ($EC_{e}$) measured in a saturated soil paste extract was $1.42\pm 0.15{\;\mathrm{dS}\;m}^{-1}$, with an initial pH of 7.7±0.1.

Soil EC During Treatment: Rhizosphere soil EC was regularly monitored during active vegetative growth stages, averaging $3.50-4.10{\;\mathrm{dS}\;m}^{-1}$ under the moderate salinity level ($S_{1}$) and $7.80-8.60{\;\mathrm{dS}\;m}^{-1}$ under the high salinity level ($S_{2}$).

Final Soil EC and pH: At final harvest, post-harvest rhizosphere soil $EC_{e}$ reached $4.50\pm 0.25{\;\mathrm{dS}\;m}^{-1}$ ($S_{1}$) and $9.20\pm 0.40{\;\mathrm{dS}\;m}^{-1}$ ($S_{2}$), compared to $1.65{\;\mathrm{dS}\;m}^{-1}$ in the control ($S_{0}$). Soil pH remained stably slightly alkaline throughout the experiment, ranging between 7.8 and 8.1.

Salinity Application Frequency: Saline water was applied at a controlled frequency of one saline irrigation out of every three consecutive irrigations to maintain target osmotic stress levels while avoiding sudden osmotic shock to the plants.

The detailed soil analysis, dynamic soil electrical conductivity monitoring, and the salinity application protocol implemented during the field experiment are presented in Supplementary Table S3.

### 3.2. Plant Material and Acclimatization

Rosemary (*Rosmarinus officinalis* L.) “Erectus; most dominant genotype in Egypt” seedlings were obtained from a certified commercial nursery. The seedlings were uniform, healthy, and pathogen-free at 8–11 cm tall. Seedlings were subjected to an acclimatization process to achieve a uniform physiological status in all experimental units before being transplanted in the field.

#### Characterization of Applied Formulations

Chitosan Nanoparticles (CsNPs): CsNPs were synthesized via ionic gelation using low-molecular-weight chitosan and sodium tripolyphosphate (TPP). TEM characterization confirmed spherical morphology, with a mean hydrodynamic diameter of 124.6±3.2 nm, a zeta potential of +34.2±1.1 mV, and a polydispersity index (PDI) of 0.22.

Seaweed Extract (Oligo-X): Oligo-X was derived from 100% natural Ascophyllum nodosum, containing 10–15% oligosaccharides, 12% alginic acid, 4–6% mannitol, 3–5% amino/fulvic acids, 600–800 ppm natural phytohormones (auxins and cytokinins), and $5–7\%\;K_{2}O$.

### 3.3. Preparation and Characterization of Chitosan Nanoparticles

Low-molecular-weight chitosan, cross-linked by sodium tripolyphosphate (TPP), was used to prepare chitosan nanoparticles (CsNPs) by ionic gelation. Dynamic Light Scattering (DLS) showed an average hydrodynamic diameter (Z-average) of 124.6 nm, and a polydispersity index (PDI) of 0.23. The surface charge measured by zeta potential was +34.2 mV, which is good for the stability of the colloid. Transmission Electron Microscopy (TEM) confirmed the spherical shape, and the core particle size was found to be between 40 and 65 nm, and there was no aggregation observed.

### 3.4. Experimental Design and Treatments

The trial was a split–split–plot randomized complete block design, with three independent blocks. Drought level was assigned to the main plots, salinity level to the sub-plots within each drought treatment, and biostimulant treatment to the sub-sub-plots within each drought × salinity combination. Thus, the treatment structure comprised 3 drought levels × 3 salinity levels × 3 biostimulant treatments = 27 treatment combinations, which were arranged in a design with 3 independent blocks, yielding a total of 81 experimental plots per harvest.

**1-Main Plot Factor—Drought Regimes (** D **—3 Levels):**

C: Control irrigation (100% Field Capacity—FC)

$D_{1}$: Moderate drought stress (70%FC)

$D_{2}$: Severe drought stress (50%FC)

**2-Sub-Plot Factor—Salinity Regimes (** S **—3 Levels):**

C: Tap water control

$S_{1}$: Moderate salinity (2850 ppm NaCl)

$S_{2}$: Severe salinity (3200 ppm NaCl)

**3-Sub-Sub-Plot Factor—Biostimulant Formulations (** B **—3 Levels):**

**Group 1 (**$B_{0}$**—Baseline Stress Matrix, 9 Treatments**): Non-stimulated control receiving distilled water spray across all drought × salinity stress combinations.

**Group 2 (**$B_{1}$**—Low Concentration Formulation, 9 Treatments**): Baseline stress matrix treated with a 1:1(v/v) blend containing 6 mL/L nano-chitosan (0.05%) + 6 mL/L *Ascophyllum nodosum* seaweed extract (Oligo-X).

**Group 3 (**$B_{2}$**—High Concentration Formulation, 9 Treatments**): Baseline stress matrix treated with a 1:1(v/v) blend containing 8 mL/L nano-chitosan (0.05%) + 8 mL/L *Ascophyllum nodosum* seaweed extract (Oligo-X).

The four seasonal harvests (H1–H4) were performed sequentially on the same experimental units, and therefore were treated as repeated measurements rather than independent replications. Drought, salinity, and biostimulant treatments were considered fixed effects, while block and plot-level variation were treated as random sources of variation. Foliar sprays were started 1 month after the imposition of stress and were made in three applications at 21-day intervals until runoff.

### 3.5. Biomass Harvesting

Harvests were conducted in four seasonal cycles: H1 (Summer 2024), H2 (Winter 2024), H3 (Summer 2025), and H4 (Winter 2025). The aboveground shoots were manually cut, leaving a uniform stubble height of 10 cm.

### 3.6. Analytical Assays, Biochemical, Physiological, and Stress Index Measurements

Measured morphological traits were: plant height, number of branches per plant, and fresh and dry biomass. Biochemical analyses included total chlorophyll [31], total carbohydrates [32], soluble sugars, tissue nitrogen [33], phosphorus [34], potassium [35], sodium [36], total phenolics [37], free proline content [38], and essential oil yield [39]. Physiological status was determined by relative water content (RWC) [40] and pressure–volume curve parameters [41].

Morphological traits like plant height (cm), primary branch count per plant, shoot fresh weight (${g\;\mathrm{plant}}^{-1}$), and shoot dry weight (${g\;\mathrm{plant}}^{-1}$, dried at 60 °C to constant weight) were recorded at each harvest.

Relative water content (RWC) was measured according to the following equation:

$$\mathrm{RWC}\;(\%)=\left(\frac{\mathrm{Fresh}\;\mathrm{Weight}-\mathrm{Dry}\;\mathrm{Weight}}{\mathrm{Turgid}\;\mathrm{Weight}-\mathrm{Dry}\;\mathrm{Weight}}\right)\times 100$$

#### 3.6.1. Biochemical and Phytochemical Assays

Total chlorophyll was extracted using 80% acetone and measured spectrophotometrically. Free proline content was estimated by acid–ninhydrin, and expressed as micromoles per grams dry weight (μmol g^−1^ DW). For determination of ion contents, the plant tissues were digested in a mixture of nitric and perchloric acids (HNO_3_: HClO_4_ 4:1, *v*/*v*), and the sodium and potassium contents were estimated by flame photometry.

#### 3.6.2. Essential Oil Analysis

Essential oil was extracted from 50 g of air-dried rosemary leaves by hydrodistillation for 3 h using a Clevenger apparatus; the separated oil was dried over anhydrous Na_2_SO_4_ and stored in sealed amber vials at 4 °C in the dark. For all 27 treatments, GC–MS analysis was performed on an Agilent 7890B/5977B system fitted with an HP-5MS (USA) column (30 m × 0.25 mm × 0.25 µm), injecting 1 µL of a 1:100 dilution in n-hexane at 250 °C (split 1:50) with helium carrier gas at 1.0 mL min^−1^, using a temperature program of 60 °C (2 min) to 200 °C at 4 °C min^−1^, then to 280 °C at 10 °C min^−1^ (hold 5 min), and MS operated in EI mode at 70 eV (*m*/*z* 40–550).

Selected treatments (the control, D1 + B2, D2 × S2, and D2 × S2 + B2) were additionally analyzed by DESI-MS on a Thermo Q Exactive Focus (USA) in positive-ion mode (spray voltage 4.5 kV, capillary 275 °C, *m*/*z* 50–500) with methanol:water (1:1, *v*/*v*) at 2 µL min^−1^. Compounds were identified by matching mass spectra to NIST 17 and Wiley 10th libraries, which were confirmed by linear retention indices (C_8_–C_30_ n-alkanes) and authentic standards (1,8-cineole, camphor, α-pinene, and camphene), and quantified as relative percentages from total ion chromatogram peak areas based on three biological replicates per treatment (n = 3), each with two technical injections.

The essential oil was obtained by hydrodistillation and the major compounds identified by gas chromatography–mass spectrometry (GC-MS) were alpha-pinene, 1,8-cineole, camphor, and verbenone.

Essential oil yield (${g\;\mathrm{plant}}^{-1}$) was calculated as:

$$\mathrm{Essential}\;\mathrm{Oil}\;\mathrm{Yield}=\mathrm{Shoot}\;\mathrm{Dry}\;\mathrm{Weight}\times \left(\frac{\mathrm{Essential}\;\mathrm{Oil}\;\%}{100}\right)$$

Essential Oil Content (% *w*/*w*): Mass of oil extracted per 100 g of dry leaf biomass ($g\;100g^{-1}\;\mathrm{DW}$).

Essential Oil Yield (${g\;\mathrm{plant}}^{-1}$): Total oil mass produced per plant, calculated as:

$$\mathrm{Essential}\;\mathrm{oil}\;\mathrm{yield}=\left(\frac{\mathrm{Essential}\;\mathrm{oil}\;\mathrm{content}\;\left(\%\right)}{100}\right)\times \mathrm{Dry}\;\mathrm{leaf}\;\mathrm{weight}\;\mathrm{per}\;\mathrm{plant}\;\left(g\right)$$

Individual Compound Abundance (% of oil): Relative peak area percentage obtained via GC-MS total ion chromatograms (TIC).

Absolute Yield of Individual Compounds (${g\;\mathrm{plant}}^{-1}$): Absolute mass production of key bioactive constituents (e.g., 1,8-cineole, camphor, and α-pinene) per plant, calculated as:

$$\begin{array}{ll} \mathrm{Absolute}\;\mathrm{yield}\;\mathrm{of} & \mathrm{compound}\;X({g\;plant}^{-1})\\ & =\left(\frac{\mathrm{Abundance}\;\mathrm{of}\;\mathrm{compound}\;X\;\left(\%\;\mathrm{of}\;\mathrm{oil}\right)}{100}\right)\\ & \times \mathrm{Essential}\;\mathrm{oil}\;\mathrm{yield}\;\left({g\;\mathrm{plant}}^{-1}\right) \end{array}$$

The complete essential oil composition for all 27 treatments in *Rosmarinus officinalis* L. is presented in Supplementary Table S7.

#### 3.6.3. Mathematical Formulations for Stress Indices

Other calculated indices were the Tolerance Index (TOL) [42], the Stress Resistance Index (SRI) [43], harvest-specific interaction scores (HIS1–6) [44,45], and the Biostimulant Efficacy Index (BEI) [46,47].

Tolerance Index (TOL): The TOL evaluates the yield reduction or loss under stress compared to favorable (non-stressed) conditions. A lower TOL value indicates smaller yield loss and higher stability across environments:

$$TOL=Y_{p}-Y_{s}$$

where $Y_{p}$ is the mean yield under non-stressed conditions and $Y_{s}$ is the yield under stress.

Stress Resistance Index (SRI): The SRI evaluates the relative yield retention capacity under stress, expressed as a percentage of the optimal yield. Higher SRI values reflect greater constitutive resilience and stress tolerance:

$$SRI=\left(\frac{Y_{s}}{Y_{p}}\right)\times 100$$

where $Y_{s}$ is the mean yield under a specific stress condition, and $Y_{p}$ is the mean yield under non-stressed control conditions.

Harvest Interaction Score ($HIS_{k}$): This score evaluates the temporal stability and yield persistence across sequential cutting/harvest cycles (k=1 to 4). It assesses whether stress tolerance changes over time or across different harvesting seasons:

$$HIS_{k}=\frac{Y_{s,k}}{Y_{p,k}}$$

where k represents the sequential harvest cut sequence (1 to 4), $Y_{s,k}$ is the yield under stress at harvest k, and $Y_{p,k}$ is the non-stressed control yield at harvest k.

Biostimulant Efficiency Index (BEI): The BEI evaluates the relative percentage improvement in a given parameter (e.g., shoot dry biomass) resulting from biostimulant application compared to the corresponding non-biostimulated (untreated) control under the same stress condition:

$$BEI\left(\%\right)=\left(\frac{Y_{\mathrm{biostimulant}}-Y_{\mathrm{untreated}}}{Y_{\mathrm{untreated}}}\right)\times 100$$

where $Y_{\mathrm{biostimulant}}$ is the observed mean value of a given trait under a specific stress level with biostimulant treatment (B1 or B2), and $Y_{\mathrm{untreated}}$ is the mean value under the same stress level without biostimulant application.

### 3.7. Statistical Analysis

A repeated-measures split–split–plot ANOVA under the General Linear Model (GLM) was performed in SPSS (v26) and R (v4.2.1) [48]. To distinguish statistical significance from biological significance, we reported the effect sizes (partial η^2^, ω^2^, and Cohen’s f) for all main effects and interactions. Partial η^2^ was calculated as SS_effect/(SS_effect + SS_error), and ω^2^ was calculated as (SS_effect − (df_effect × MS_error))/(SS_total + MS_error). Cohen’s f was derived as √(η^2^/(1 − η^2^)). For treatment means, we report standard errors and 95% confidence intervals (CIs). For key pairwise contrasts, we report the mean difference with 95% CIs. The Least Significant Difference (LSD) test was used for mean separation at *p* < 0.05 [49], but biological interpretation was based on effect sizes and CIs rather than *p*-values alone. All effect sizes and CIs are provided in Supplementary Table S5. Multivariate analysis was performed using MANOVA [50], Principal Component Analysis (PCA) [51], and hierarchical clustering based on Ward’s method and squared Euclidean distances [52].

Data collected across the 4 sequential harvests ($H_{1}–H_{4}$) from the same permanent $3.0{\;m}^{2}$ experimental units were subjected to a repeated-measures Analysis of Variance (RM-ANOVA) based on a 3×3×3 arrangement in a split–split–plot design with 3 replicates (blocks). Drought regimes (D), salinity regimes (S), and biostimulant formulations (B) served as fixed main factors, while harvest cut (H) was designated as a repeated-measures split-in-time factor. Block effects were incorporated into the model to control spatial variability.

## 4. Conclusions

The results of this study show that rosemary has high intrinsic stress tolerance, making it well-suited for low-input sustainable horticulture in stress-prone arid regions. Under severe combined stress ($D_{2}\times S_{2}$), biostimulants (especially B2) improved tissue hydration and ionic balance ($K^{+}/Na^{+}$ ratio), and preserved high phenotypic stability without significant physiological collapse. However, exogenous nano-biostimulants are not essential for basal survival, but act as stress modulators; their application is most effective under moderate drought ($D_{1}$) to optimize essential oil quality and key monoterpenes. Thus, biostimulants should be viewed as an optional management tool for quality enhancement rather than an emergency remedy for extreme stress. A limitation of this work is that carbon allocation and photosynthetic rate were not directly measured. Future studies should investigate carbon partitioning and photosynthetic efficiency under combined stress to clarify the metabolic costs of biostimulant-mediated stress acclimation.

## Figures and Tables

**Figure 1:**
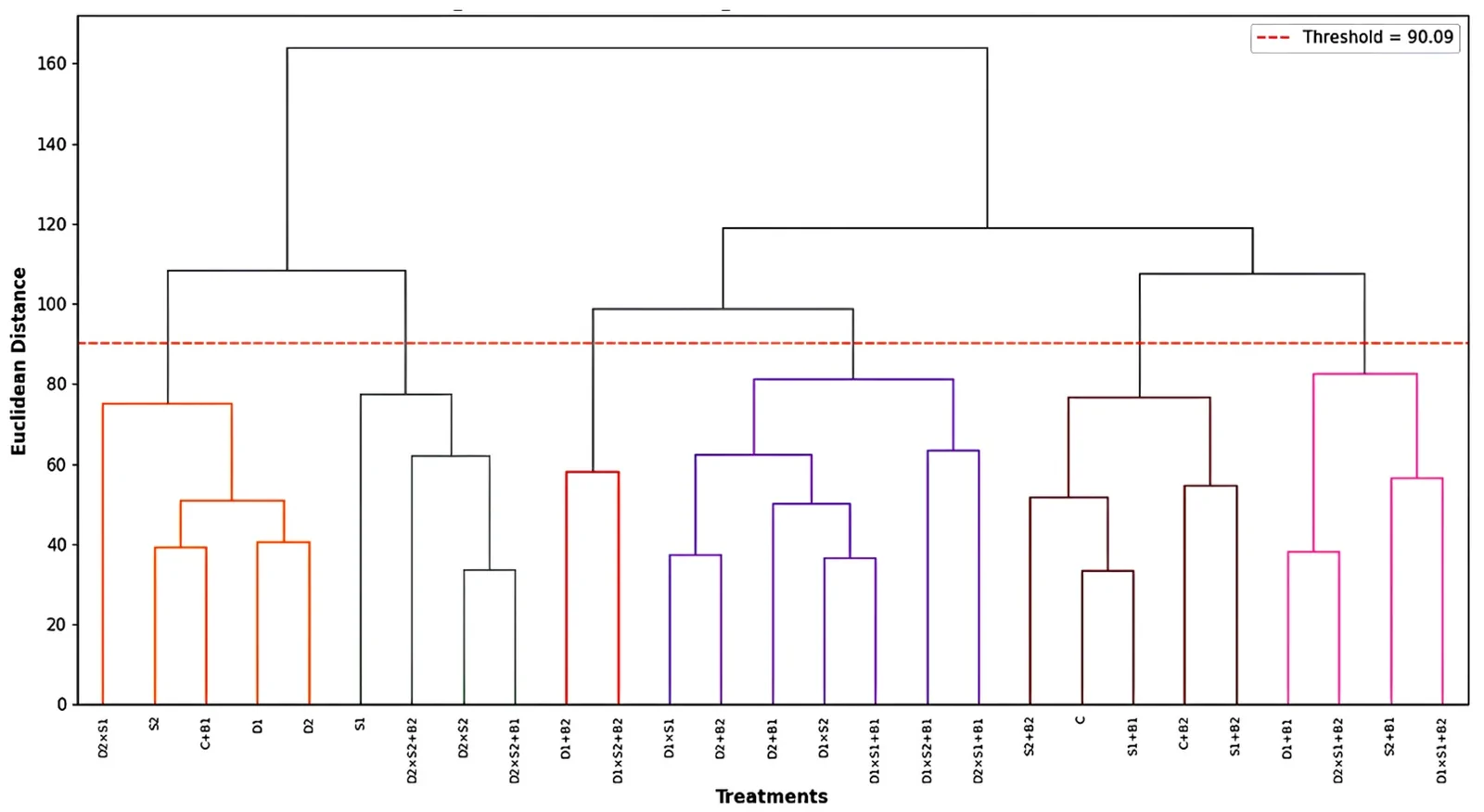
Hierarchical cluster dendrogram illustrating structural relationships among twenty-seven *Rosmarinus officinalis* L. treatment combinations based on squared Euclidean distances.

**Figure 2:**
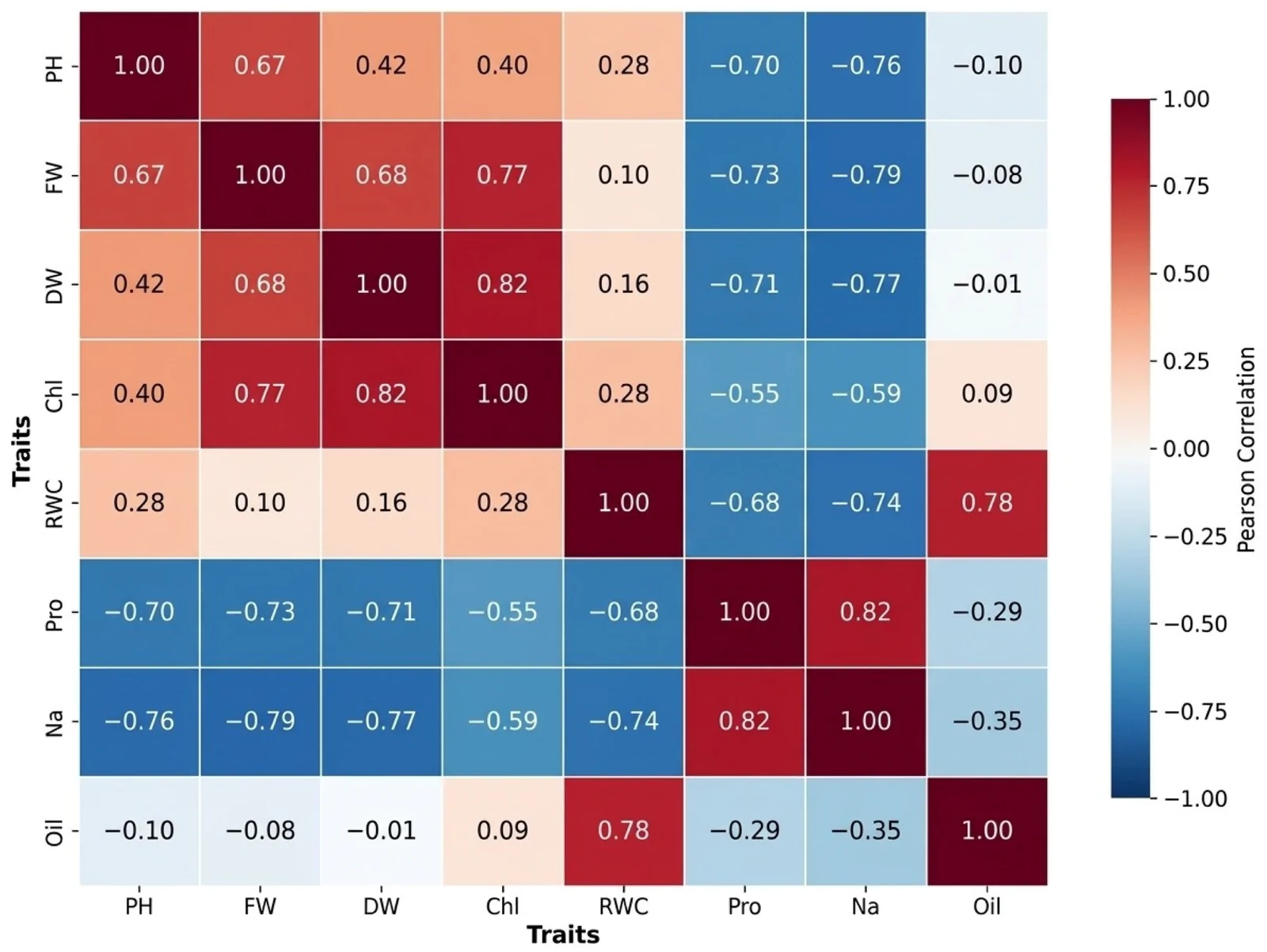
Pearson correlation matrix heat map representation for key agronomic, physiological, and stress-responsive traits in *Rosmarinus officinalis* L., where abbreviations denote: PH = plant height, FW = fresh weight, DW = dry weight, Chl = chlorophyll, RWC = relative water content, Pro = proline, Na = sodium, and Oil = essential oil.

**Figure 3:**
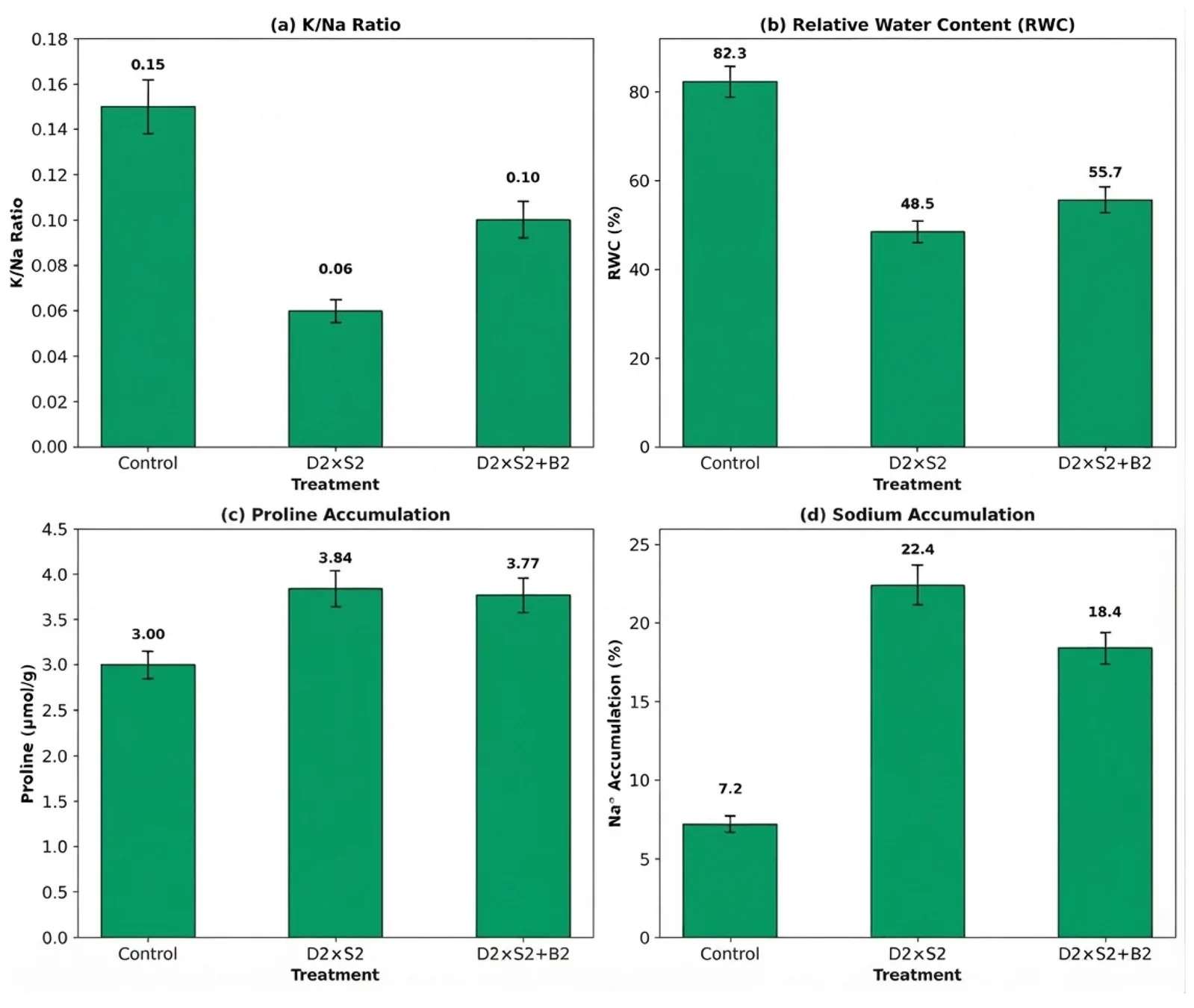
Ion homeostasis and physiological adjustments in *Rosmarinus officinalis* L. under severe combined stress ($D_{2}\times S_{2}$): (**a**) Potassium-to-sodium ratio ($K^{+}/Na^{+}$), (**b**) relative water content (RWC), (**c**) proline accumulation, and (**d**) sodium ($Na^{+}$) accumulation.

**Figure 4:**
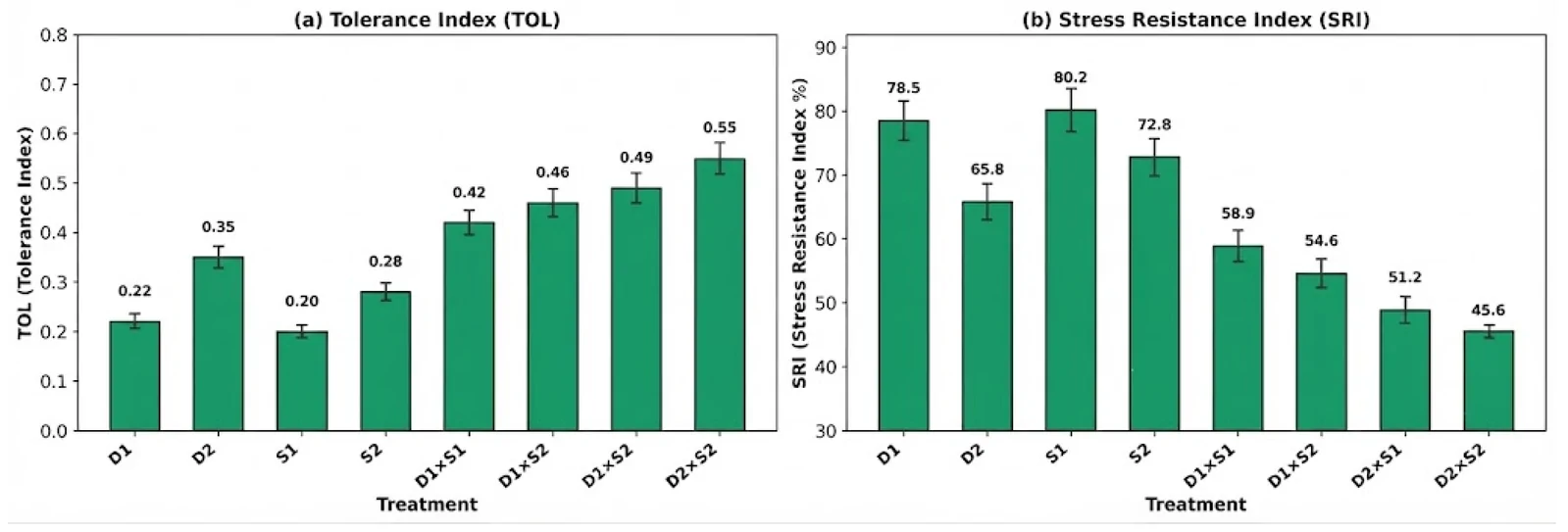
Tolerance indices for *Rosmarinus officinalis* L.: (**a**) Tolerance Index (TOL); (**b**) Stress Resistance Index (SRI).

**Figure 5:**
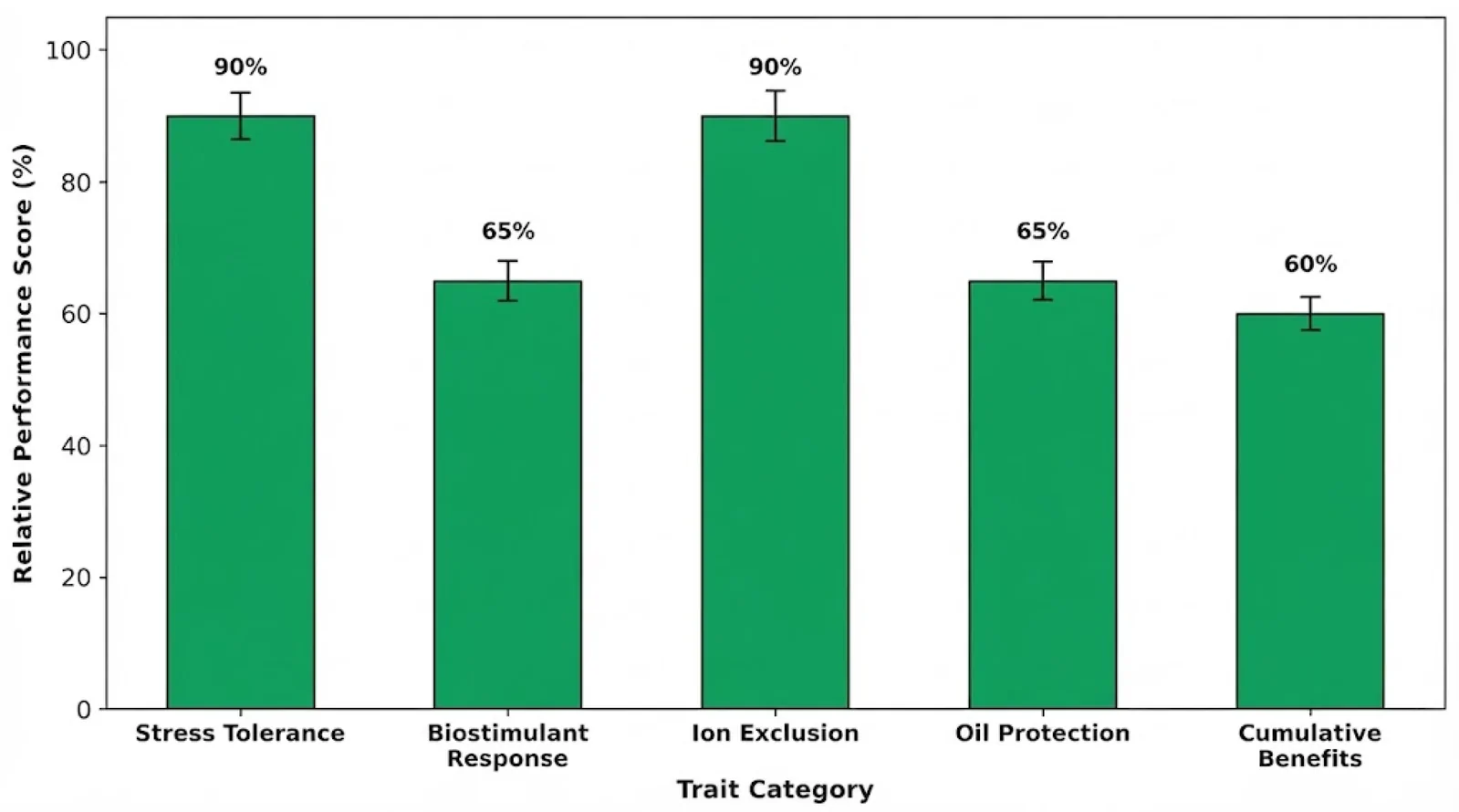
Biostimulant Efficiency Index (BEI) dynamics across sequential cutting cycles ($H_{1}$ to $H_{4}$) in *Rosmarinus officinalis* L.

**Table 1:** Summary of the repeated-measures split–split–plot ANOVA for the most significant morphological, physiological, and biochemical traits of *Rosmarinus officinalis* L. (F-values).

Trait	Year (Y)	Cut (C)	Treatment (T)	Y × C	Y × T	C × T	Y × C × T	CV%
Plant Height	78.34 ***	189.67 ***	60.23 ***	28.12 ***	7.28 ***	9.05 ***	3.02 ***	7.89
Branch Number	42.34 ***	98.56 ***	35.67 ***	16.78 ***	5.12 ***	6.34 ***	2.56 ***	8.89
Fresh Weight	72.34 ***	145.67 ***	48.90 ***	22.34 ***	6.78 ***	7.89 ***	2.89 ***	9.45
Dry Weight	52.34 ***	122.34 ***	41.23 ***	19.56 ***	5.89 ***	6.78 ***	2.45 ***	8.89
Chlorophyll	31.23 ***	82.34 ***	26.45 ***	11.23 ***	3.89 ***	4.67 ***	1.89 ***	5.12
RWC	26.78 ***	68.90 ***	23.89 ***	9.89 ***	3.34 ***	4.01 ***	1.62 ***	5.01
Oil (%)	23.56 ***	58.90 ***	19.23 ***	8.56 ***	3.12 ***	3.89 ***	1.56 ***	6.23
Proline	38.90 ***	94.56 ***	32.34 ***	14.56 ***	4.45 ***	5.23 ***	2.01 ***	8.01
Sodium	35.67 ***	88.90 ***	29.89 ***	13.45 ***	4.12 ***	4.89 ***	1.89 ***	8.89
K/Na Ratio	28.34 ***	72.45 ***	24.89 ***	10.56 ***	3.51 ***	4.21 ***	1.63 ***	6.78

*** *p* < 0.001; the complete ANOVA is provided in Supplementary Table S2.

**Table 2:** Trait responses of *Rosmarinus officinalis* L. using Multivariate Analysis of Variance (MANOVA).

Effect	Wilks’ λ	F-Value	df1	df2	*p*-Value	Partial η^2^
**Year**	0.289	78.34	9	46	<0.001	0.878
**Cut**	0.212	189.67	27	154	<0.001	0.912
**Treatment**	0.056	60.23	234	46	<0.001	0.889
**Year × Cut**	0.423	28.12	27	154	<0.001	0.712
**Year × Treatment**	0.445	7.28	234	46	<0.001	0.612
**Cut × Treatment**	0.423	9.05	702	154	<0.001	0.598
**Year × Cut × Treatment**	0.512	3.02	702	154	<0.001	0.487

Note: ${\mathrm{df}}_{1}$ and ${\mathrm{df}}_{2}$ represent the hypothesis and error degrees of freedom, respectively, calculated for Wilks’ Lambda (λ) using Rao’s F-approximation to account for the multivariate trait vectors and the hierarchical split–split–plot repeated-measures design.

**Table 3:** Selected treatment means for key *Rosmarinus officinalis* L. traits.

Treatment	PH (cm)	FW (g)	DW (g)	Chl (mg/g)	Oil (%)	RWC (%)	Pro (μmol/g)	Na (%)	K/Na
**C**	39.23 ^b^	73.45 ^b^	18.34 ^b^	1.89 ^b^	0.37 ^b^	82.34 ^b^	3.00 ^e^	7.20 ^e^	0.15 ^a^
**D2 × S2**	22.78 ^f^	49.23 ^f^	12.23 ^f^	1.32 ^e^	0.15 ^f^	48.45 ^j^	3.84 ^d^	22.40 ^a^	0.06 ^f^
**D2 × S2 + B1**	22.12 ^g^	48.45 ^f^	11.89 ^g^	1.33 ^e^	0.22 ^e^	52.34 ^i^	3.77 ^d^	20.80 ^b^	0.08 ^d^
**D2 × S2 + B2**	21.78 ^g^	50.12 ^f^	11.89 ^g^	1.32 ^e^	0.22 ^e^	55.67 ^h^	3.77 ^d^	18.40 ^c^	0.10 ^c^
**C + B2**	54.12 ^a^	110.23 ^a^	27.34 ^a^	2.68 ^a^	0.41 ^a^	92.34 ^a^	3.20 ^e^	5.80 ^f^	0.20 ^b^
**LSD (0.05)**	2.18	5.45	2.34	0.23	0.08	2.18	0.24	1.10	0.03

Different letters indicate significant differences at *p* ≤ 0.05. Means sharing the same letter within a column do not differ significantly at p≤0.05 according to the LSD test. PH: plant height; FW: fresh weight; DW: dry weight; Chl: total chlorophyll; Oil: essential oil percentage; RWC: relative water content; and Pro: free proline.

**Table 4:** Key correlation findings.

Relationship	Correlation Coefficient (r)	Interpretation
FW ↔ DW	r=0.68	Strong positive correlation indicating consistent biomass allocation.
Oil ↔ RWC	r=0.78	Strong association between water and oil production, indicating the dependence of essential oil production on tissue water potential.
Na ↔ DW	r=−0.77	The toxicity of sodium had a great influence on biomass production.
Pro ↔ DW	r=−0.71	Accumulation of proline, which can be used as a reliable marker of stress.
K/Na ↔ RWC	r=0.72	Critical ion homeostasis–water status link that may be indicative of osmotic adjustment and membrane protection.

**Table 5:** Ion homeostasis and hydration parameters under severe combined stress ($D_{2}\times S_{2}$) and biostimulant rescue ($D_{2}\times S_{2}+B_{2}$).

Parameter	Control	D2 × S2	D2 × S2 + B2	Improvement
RWC (%)	82.34	48.45	55.67	+14.9%
K/Na Ratio	0.15	0.06	0.10	+66.7%
Na^+^ Accumulation (%)	7.20	22.40	18.40	−17.9%
Proline (μmol/g DW)	3.00	3.84	3.77	−1.8%

**Table 6:** Derived stress tolerance indices for *Rosmarinus officinalis* L. across stress and biostimulant regimes.

Treatment Regime	Tolerance Index (TOL)	Stress Resistance Index (SRI)	Harvest Score ($HIS_{6}$)
$D_{1}$ (70% FC)	0.22	78.5	0.78
$D_{2}$ (50% FC)	0.35	65.8	0.72
$S_{1}$ (2850 ppm NaCl)	0.20	80.2	0.80
$S_{2}$ (3200 ppm NaCl)	0.28	72.8	0.75
$D_{1}\times S_{1}$	0.42	58.9	0.62
$D_{1}\times S_{2}$	0.46	54.6	0.58
$D_{2}\times S_{1}$	0.49	51.2	0.55
$D_{2}\times S_{2}$	0.55	45.6	0.45
$D_{2}\times S_{2}+B_{2}$	0.52	48.5	0.52
$C+B_{2}$	0.16	84.2	0.88

**Table 7:** Biostimulant Efficiency Index (BEI %) for *Rosmarinus officinalis* L. across four sequential harvest cuts.

Treatment	Harvest 1 ($H_{1}$)	Harvest 2 ($H_{2}$)	Harvest 3 ($H_{3}$)	Harvest 4 ($H_{4}$)	Mean BEI (%)
Formulation $B_{1}$:					
$D_{1}+B_{1}$	11.2%	13.8%	16.5%	15.2%	14.2%
$D_{2}+B_{1}$	7.5%	9.8%	11.8%	10.5%	9.9%
$S_{1}+B_{1}$	9.8%	11.5%	14.2%	12.8%	12.1%
$S_{2}+B_{1}$	5.2%	6.8%	8.5%	7.5%	7.0%
Mean $B_{1}$	8.5%	10.2%	12.8%	11.5%	10.8%
Formulation $B_{2}$:					
$D_{1}+B_{2}$	16.8%	20.5%	23.8%	22.2%	20.8%
$D_{2}+B_{2}$	11.5%	14.2%	17.2%	15.8%	14.7%
$S_{1}+B_{2}$	14.2%	17.5%	21.2%	19.5%	18.1%
$S_{2}+B_{2}$	8.5%	11.2%	13.8%	12.5%	11.5%
Mean $B_{2}$	13.2%	15.8%	18.5%	17.0%	16.1%
$B_{2}$ Advantage	+4.7%	+5.6%	+5.7%	+5.5%	+5.3%

**Table 8:** Cumulative effects across years and seasons (*Rosmarinus officinalis* L).

Parameter	2024	2025	Summer	Winter
Plant Height (cm)	34.5 ± 2.8	38.2 ± 3.1	39.5 ± 3.2	33.2 ± 2.5
Fresh Weight (g)	65.8 ± 4.5	72.3 ± 5.2	75.8 ± 5.5	62.3 ± 4.2
Dry Weight (g)	16.8 ± 1.2	18.5 ± 1.4	19.2 ± 1.5	16.1 ± 1.1
Oil (%)	0.26 ± 0.02	0.30 ± 0.03	0.31 ± 0.03	0.25 ± 0.02
TPC (mg/g)	65.8 ± 3.2	69.4 ± 3.5	68.5 ± 3.4	63.2 ± 3.0

**Table 9:** Essential oil composition (%) of *Rosmarinus officinalis* L. under selected treatments.

Treatment	α-Pinene (%)	1,8-Cineole (%)	Camphor (%)	Verbenone (%)
C	19.66	7.84	33.50	6.70
D1	22.68	8.38	40.44	8.50
D2	14.00	7.12	22.50	5.75
S1	21.00	8.08	36.00	7.50
S2	14.20	7.30	26.40	5.80
D1 × S1	13.77	6.72	14.20	5.60
D2 × S2	12.66	6.21	6.89	5.14
S1 + B2	21.80	8.16	40.00	7.86
D1 + B1	27.92	9.00	41.00	8.80
D1 + B2	28.14	9.86	42.14	9.03
D2 × S2 + B2	13.00	6.24	6.92	5.18

## Data Availability

All data generated or analyzed during this study are included in this published article and its supplementary information files. The data that support the findings of this study are available from the corresponding author upon reasonable request.

## Supplementary Materials

The following supporting information can be downloaded at: https://www.mdpi.com/article/10.3390/plants15192919/s1, Figure S1: DESI-MS mass spectrometric profiles of rosemary essential oil. Comparative mass spectra showing the relative abundance of key volatile constituents (α-pinene, camphor, and 1,8-cineole) under the control, biostimulated (D1 + B2), and severe stress (D2 × S2) conditions; Figure S2: Spatial distribution heatmaps of camphor (*m*/*z* 153.13). MS imaging tiles illustrating the high-intensity distribution of camphor in the control and recovery samples in contrast to the significant signal decline observed under severe stress conditions; Figure S3: Mass spectra and chemical structures of major rosemary volatile constituents. Detailed fragmentation patterns and molecular ion peaks (M^+^) for camphor (*m*/*z* 152.12), α-pinene (*m*/*z* 136.12), and 1,8-cineole (*m*/*z* 154.13) employed for compound identification and structural confirmation; Figure S4: Quantitative comparison of volatile compounds and chemical class profiling. (A) Bar chart representing the percentage of total oil for primary compounds across experimental treatments; (B) heatmap matrix of quantitative percentage distributions; and (C) stacked bar chart illustrating the relative distribution of oxygenated monoterpenes, monoterpene hydrocarbons, and sesquiterpenes across treatment groups.; Table S1: Chronological harvest cycles and corresponding meteorological conditions at Arish University (2024–2025); Table S2: Baseline physico-chemical properties of the experimental soil prior to land preparation and fertilization; Table S3: Soil characteristics, dynamic EC monitoring, and salinity application protocol; Table S4: Complete treatment means for all 27 traits in Rosmarinus officinalis L.; Table S5: Complete ANOVA summary (SS, MS, F, and P) for all 27 traits in *Rosmarinus officinalis* L.; Table S6: Effect sizes (partial η^2^, ω^2^, Cohen’s f) for ANOVA effects and 95% confidence intervals for key treatment contrasts in *Rosmarinus officinalis* L; and Table S7: Complete essential oil composition for all 27 treatments in *Rosmarinus officinalis* L.

## Abbreviations

The following abbreviations are used in this manuscript:

ANOVA	Analysis of Variance
B_1_	Biostimulant Blend 1
B_2_	Biostimulant Blend 2
BEI	Biostimulant Efficacy Index
C	Control
Chl	Chlorophyll
CsNPs	Chitosan Nanoparticles
*CV*	Coefficient of Variation
D_1_	Moderate Drought Stress
D_2_	Severe Drought Stress
DESI-MS	Desorption Electrospray Ionization Mass Spectrometry
df	Degrees of Freedom
DLS	Dynamic Light Scattering
DW	Dry Weight
EO%	Essential Oil Percentage
FW	Fresh Weight
GC-MS	Gas Chromatography–Mass Spectrometry
GLM	General Linear Model
H_1_	Harvest 1
H_2_	Harvest 2
H_3_	Harvest 3
H_4_	Harvest 4
HIS	Harvest Interaction Indices (HIS_1−6_)
KV	Kilovolt
LSD	Least Significant Difference
mL/L	Milliliters per Liter
MANOVA	Multivariate Analysis of Variance
MAPs	Medicinal and Aromatic Plants
mV	Millivolt
p	Probability
PCA	Principal Component Analysis
PH	Plant Height
Pro	Proline
PWC	Pressure–Volume Water (Parameters)
RCBD	Randomized Complete Block Design
RWC	Relative Water Content
S_1_	Moderate Salinity Stress
S_2_	Severe Salinity Stress
SRI	Stress Resistance Index
T	Treatment
TEM	Transmission Electron Microscopy
TOL	Tolerance Index
TPC	Total Phenolic Content
TPP	Tripolyphosphate
η^2^	Eta Squared
