# Supplementary material for: Physiological and Biochemical Resilience of Rosemary to Combined Drought and Salinity: A Two-Year Study of Biostimulant-Mediated Stress Mitigation
Source: Plants (Basel). 2026 Sep 23;15(19):2919. doi: 10.3390/plants15192919 (PMC13667224; doi:10.3390/plants15192919)
Supplement: Supplementary file 1 [file plants-15-02919-s001.zip › plants-4542209-supplementary.pdf]

SUPPLEMANTARY MATERIALS

1. Supplementary Figures

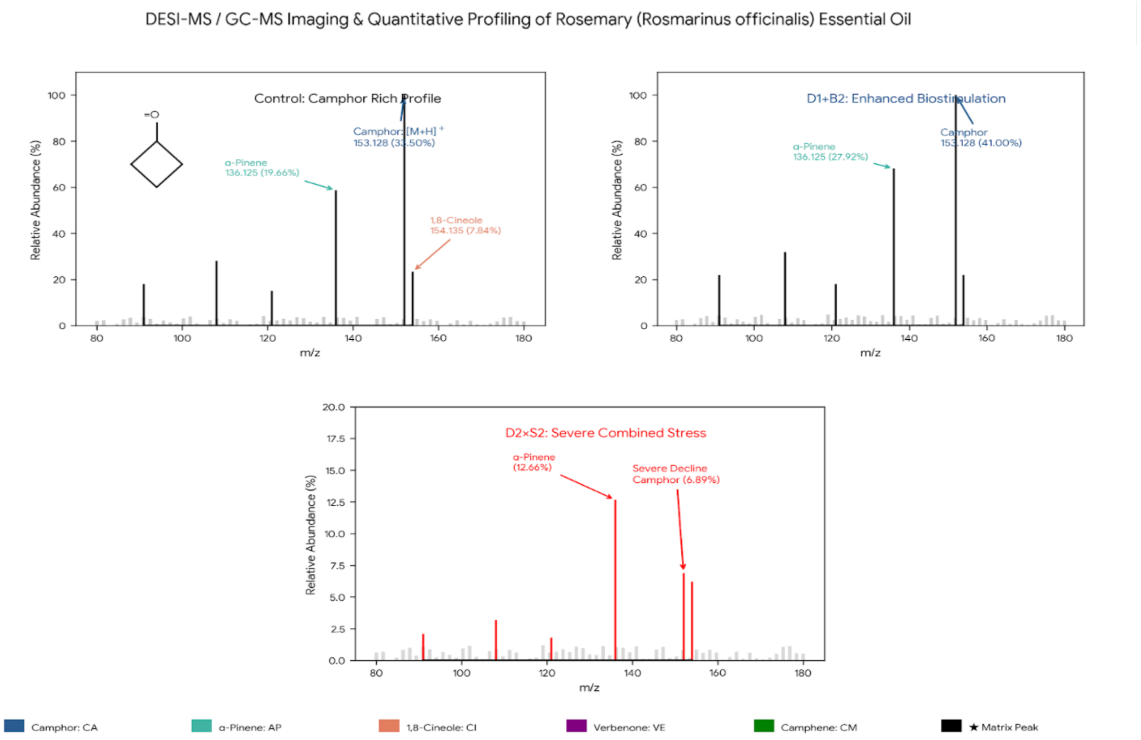

Figure S1. DESI-MS mass spectrometric profiles of rosemary essential oil. Comparative mass spectra showing the relative abundance of key volatile constituents (α-pinene, camphor, and 1,8-cineole) under control, biostimulated (D1+B2), and severe stress (D2×S2) conditions.

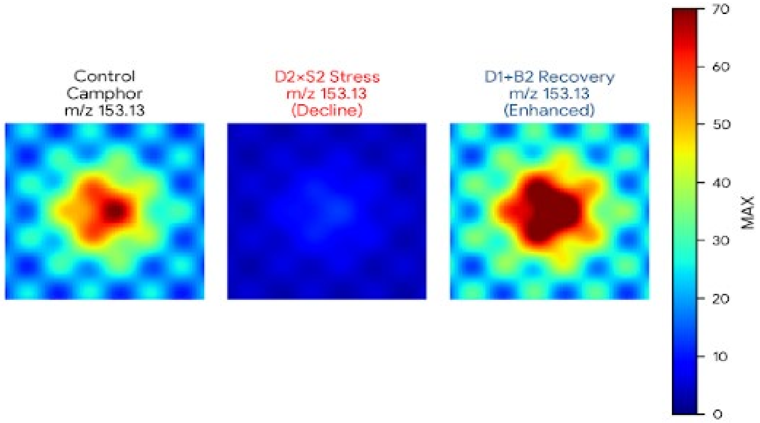

Figure S2. Spatial distribution heatmaps of camphor (m/z 153.13). MS imaging tiles illustrating the high-intensity distribution of camphor in control and recovery samples in contrast to the significant signal decline observed under severe stress conditions.

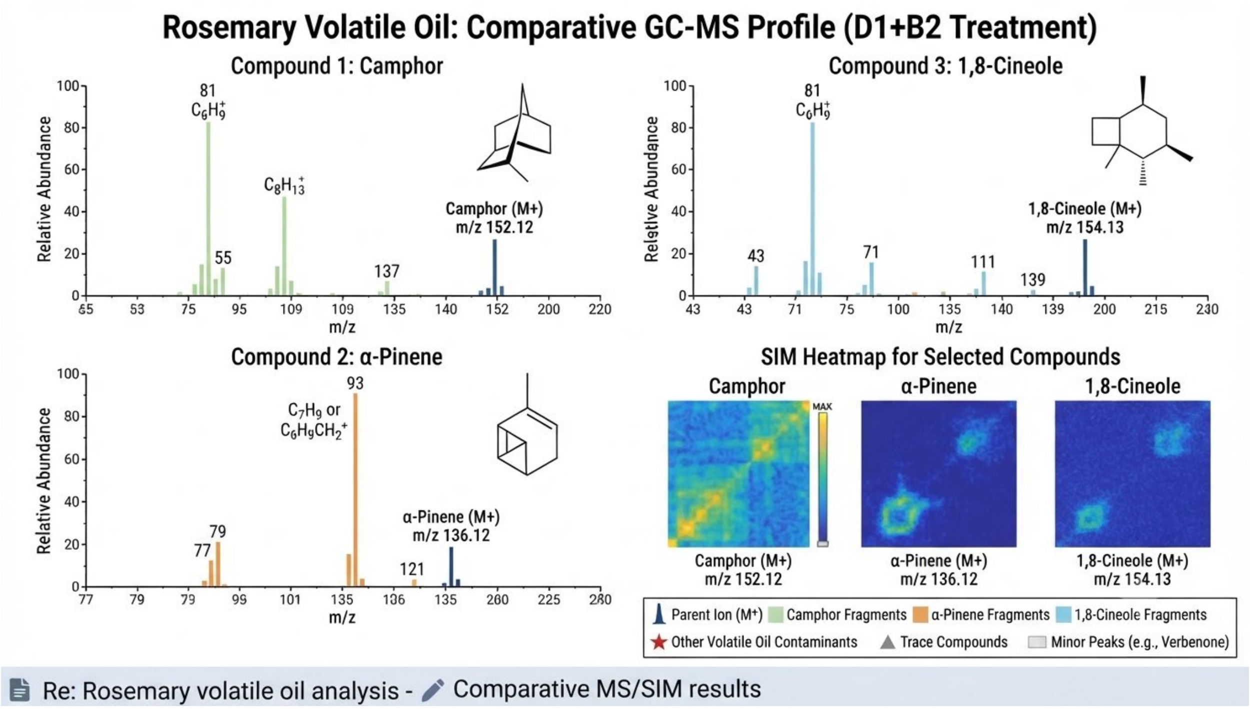

Figure S3. Mass spectra and chemical structures of major rosemary volatile constituents. Detailed fragmentation patterns and molecular ion peaks (M^+) for camphor (m/z 152.12), α-pinene (m/z 136.12), and 1,8-cineole (m/z 154.13) employed for compound identification and structural confirmation.

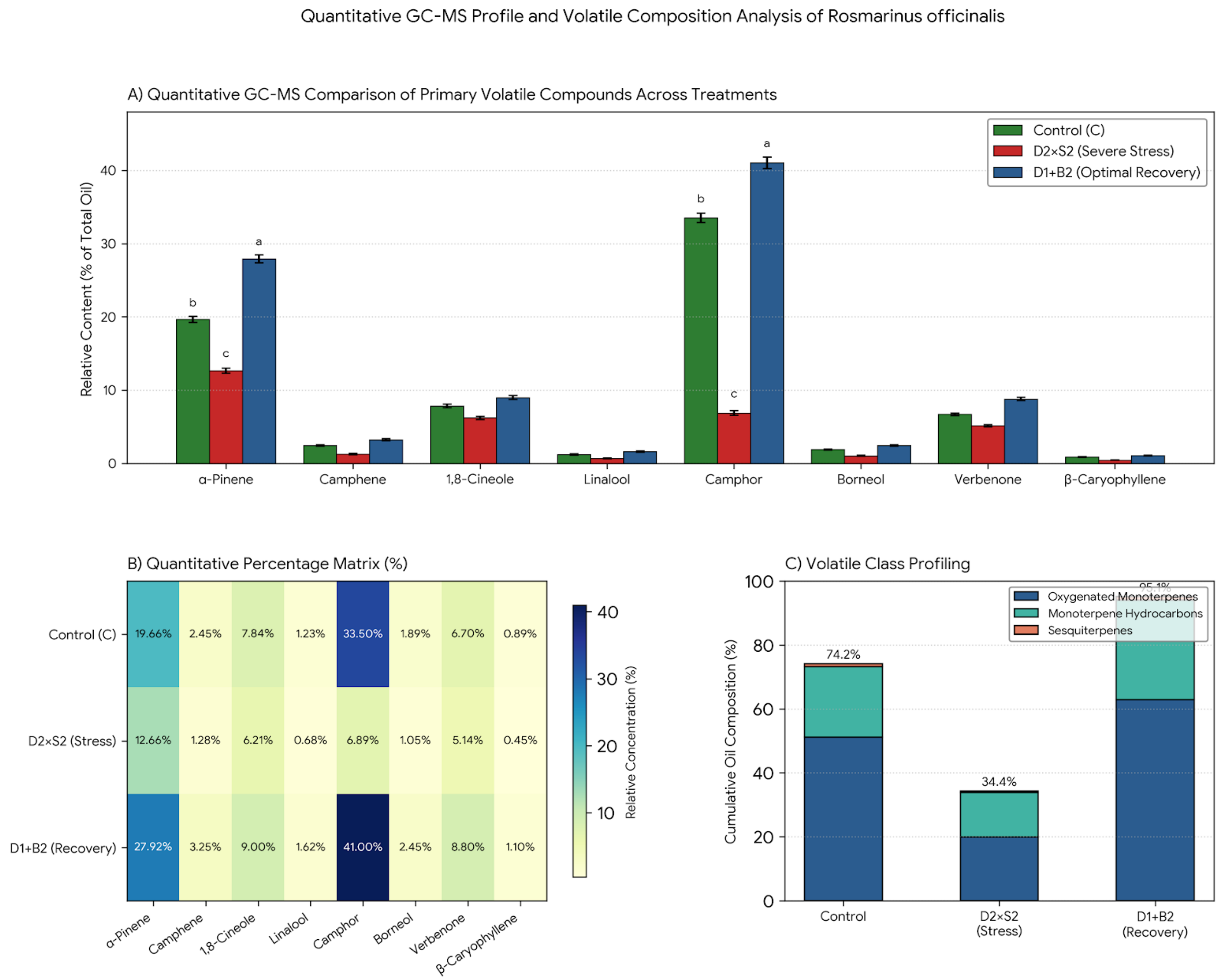

Figure S4. Quantitative comparison of volatile compounds and chemical class profiling. (A) Bar chart representing the percentage of total oil for primary compounds across experimental treatments; (B) Heatmap matrix of quantitative percentage distributions; (C) Stacked bar chart illustrating the relative distribution of oxygenated monoterpenes, monoterpene hydrocarbons, and sesquiterpenes across treatment groups.

2. Supplementary Tables

Table S1: Chronological Harvest Cycles and Corresponding Meteorological Conditions at Arish University (2024–2025)

Harvest Cycle	Season & Year	Temperature Range (Max/Min °C)	Mean Relative Humidity (%)
**Cut 1**	Winter, Feb 2024	19.5 – 22.0 / 9.0 – 11.5	65 – 70
**Cut 2**	Summer, Aug 2024	31.0 – 33.5 / 22.5 – 24.5	72 – 78
**Cut 3**	Winter, Feb 2025	18.5 – 21.5 / 8.5 – 11.0	68 – 74
**Cut 4**	Summer, Aug 2025	32.0 – 34.5 / 23.0 – 25.0	70 – 76

Data Source: Meteorological data were provided by the Central Laboratory for Agricultural Climate (CLAC), Agricultural Research Center, Egypt.

Harvesting Protocol: Shoots were clipped at a 10 cm residual height for all four cycles

Table S2: Baseline physicochemical properties of the experimental soil prior to land preparation and fertilization.

Soil Property	Unit	Value
**Physical Properties**		
Sand	%	88.5

Soil Property	Unit	Value
Silt	%	7.2
Clay	%	4.3
**Soil Texture**	—	**Sandy**
**Chemical Properties**		
pH (1:2.5 soil/water)	—	8.10
EC (1:2.5 extract)	dS m^{-1}	1.95
Organic Matter (OM)	%	0.42
$CaCO_3$	%	7.5
Available N	mg kg^{-1}	22.0
Available P	mg kg^{-1}	8.0
Available K	mg kg^{-1}	140.0

- **Data Validity:** Soil samples were collected from the 0–30 cm topsoil layer across the experimental site using a systematic grid sampling pattern.
- **Methodological Note:** These values represent the **pristine (baseline) soil characteristics** before any mechanical tillage, organic amendment incorporation (chicken manure), or chemical fertilization (mono-superphosphate, potassium sulfate, and calcium sulfate) were applied.

- **Analysis:** Analyses were performed according to standard soil science protocols for arid sandy environments to provide an objective basis for evaluating plant responses to experimental stress factors.

Table S3. Soil characteristics, dynamic EC monitoring, and salinity application protocol

Soil & Salinity Parameters	Value / Description	Measurement Method / Notes
Initial Soil Physical Properties		Hydrometer method
Sand (%)	86.4 ± 1.2	Sandy soil texture typical of the Arish region
Silt (%)	8.2 ± 0.8	
Clay (%)	5.4 ± 0.5	
Initial Soil Chemical Properties (Pre-treatment)		
Initial Soil EC_e ($dS\ m^{-1}$)	1.42 ± 0.15	Measured in saturated soil paste extract

Initial Soil pH	7.7 ± 0.1	Measured in 1:2.5 soil suspension
Soil EC During Active Growth Stages		Regular monitoring in the rhizosphere
Moderate Salinity (S_1)	3.50 – 4.10	Mid and late vegetative growth phases
High Salinity (S_2)	7.80 – 8.60	Mid and late vegetative growth phases
Final Soil EC at Harvest (EC_e)		Post-harvest rhizosphere analysis
Control (S_0)	1.65 ± 0.10	Fresh water irrigation
Moderate Salinity (S_1)	4.50 ± 0.25	Accumulation at final harvest
High Salinity (S_2)	9.20 ± 0.40	Accumulation at final harvest
Soil pH Range (Throughout Treatment)	7.8 – 8.1	Stable slight alkalinity across all plots

Salinity Application Protocol		
— Irrigation Water Source	Saline groundwater / Mixed water	Typical of North Sinai agricultural wells
— Frequency of Application	One saline irrigation out of every three consecutive irrigations	Imposed to maintain target osmotic stress and prevent sudden osmotic shock

Table S4: Complete Treatment Means for All 27 Traits in *Rosmarinus officinalis* L.

Treatment	PH (cm)	BN	FW (g)	DW (g)	Chl (mg/g)	Car (mg/g)	Carb (%)	SS (mg/g)	TPC (mg/g)	N (%)	P (%)	K (%)	Na (%)	C/N	Pro (µmol/g)	Oil (%)	RWC (%)	PWC (%)	TOL	SRI	BEI	Prot (%)	K/Na
**C**	39.23 ±2.10	9.5 ±0.40	73.45 ±3.20	18.34 ±1.10	1.89 ±0.05	0.95 ±0.03	48.2 ±1.8	16.5 ±0.7	71.23 ±2.5	2.12 ±0.07	0.32 ±0.01	0.92 ±0.03	7.20 ±0.4	8.95 ±0.4	3.00 ±0.08	0.37 ±0.02	82.34 ±2.0	72.45 ±1.8	0.28 ±0.01	72.3 ±1.5	1.00 ±0.00	13.2 ±0.5	0.15 ±0.01
**D1**	31.78 ±1.80	7.5 ±0.30	65.23 ±2.80	15.67 ±0.90	1.62 ±0.04	0.85 ±0.02	42.5 ±1.5	20.8 ±0.8	76.23 ±2.8	1.98 ±0.06	0.28 ±0.01	0.85 ±0.03	9.80 ±0.5	9.45 ±0.4	3.98 ±0.12	0.38 ±0.02	75.67 ±1.8	68.12 ±1.6	0.28 ±0.01	72.3 ±1.5	0.00 ±0.00	12.4 ±0.4	0.10 ±0.01
**D2**	29.12 ±1.60	7.0 ±0.30	60.78 ±2.50	15.12 ±0.85	1.57 ±0.04	0.82 ±0.02	38.8 ±1.3	23.5 ±0.9	63.12 ±2.2	1.85 ±0.06	0.25 ±0.01	0.78 ±0.02	12.40 ±0.6	10.12 ±0.5	4.02 ±0.12	0.25 ±0.01	68.45 ±1.6	60.12 ±1.4	0.42 ±0.02	58.2 ±1.8	0.00 ±0.00	11.6 ±0.4	0.08 ±0.01
**S1**	36.45 ±2.00	9.0 ±0.40	70.23 ±3.00	17.34 ±1.00	1.80 ±0.05	0.88 ±0.03	45.5 ±1.6	18.2 ±0.7	78.23 ±2.8	2.05 ±0.07	0.30 ±0.01	0.88 ±0.03	8.50 ±0.4	8.45 ±0.3	3.00 ±0.08	0.39 ±0.02	79.23 ±1.8	70.12 ±1.6	0.25 ±0.01	75.6 ±1.6	0.00 ±0.00	12.8 ±0.4	0.12 ±0.01
**S2**	34.12 ±1.90	9.0 ±0.40	68.45 ±2.90	17.12 ±1.00	1.62 ±0.04	0.86 ±0.03	40.2 ±1.4	22.5 ±0.8	64.23 ±2.3	1.88 ±0.06	0.26 ±0.01	0.82 ±0.02	10.80 ±0.5	9.45 ±0.4	3.00 ±0.08	0.15 ±0.01	72.34 ±1.8	62.45 ±1.4	0.32 ±0.01	68.4 ±1.5	0.00 ±0.00	11.8 ±0.4	0.08 ±0.01
**D1×S1**	24.56 ±1.40	7.5 ±0.30	50.23 ±2.20	12.45 ±0.70	1.40 ±0.04	0.72 ±0.02	35.5 ±1.2	26.8 ±0.9	35.12 ±1.5	1.52 ±0.05	0.22 ±0.01	0.65 ±0.02	16.80 ±0.8	11.45 ±0.5	3.94 ±0.12	0.15 ±0.01	58.34 ±1.6	52.34 ±1.4	0.48 ±0.02	52.3 ±1.8	0.00 ±0.00	9.5 ±0.3	0.06 ±0.01
**D1×S2**	23.34 ±1.30	7.3 ±0.30	48.78 ±2.10	12.12 ±0.70	1.34 ±0.04	0.69 ±0.02	33.8 ±1.1	28.5 ±0.9	33.89 ±1.4	1.48 ±0.05	0.20 ±0.01	0.62 ±0.02	18.20 ±0.8	11.80 ±0.5	3.91 ±0.12	0.15 ±0.01	55.67 ±1.5	50.12 ±1.3	0.52 ±0.02	48.5 ±1.6	0.00 ±0.00	9.2 ±0.3	0.05 ±0.01
**D2×S1**	21.45 ±1.20	6.5 ±0.30	45.67 ±2.00	11.34 ±0.65	1.33 ±0.03	0.67 ±0.02	31.5 ±1.0	29.5 ±0.9	35.12 ±1.4	1.42 ±0.04	0.18 ±0.01	0.58 ±0.02	19.80 ±0.9	12.10 ±0.5	3.84 ±0.11	0.15 ±0.01	52.34 ±1.4	46.78 ±1.2	0.55 ±0.02	45.8 ±1.5	0.00 ±0.00	8.9 ±0.3	0.04 ±0.01

**D2×S2**	22.78 ±1.20	6.2 ±0.30	49.23 ±2.10	12.23 ±0.70	1.32 ±0.03	0.68 ±0.02	30.2 ±1.0	31.2 ±0.9	36.12 ±1.5	1.38 ±0.04	0.16 ±0.01	0.55 ±0.02	22.40 ±0.9	12.45 ±0.5	3.84 ±0.11	0.15 ±0.01	48.45 ±1.4	42.34 ±1.2	0.62 ±0.02	38.9 ±1.4	0.00 ±0.00	8.6 ±0.3	0.04 ±0.01
**C+B1**	46.78 ±2.40	11.5 ±0.50	90.23 ±3.80	22.56 ±1.30	2.50 ±0.07	1.10 ±0.04	52.5 ±1.8	14.5 ±0.6	72.23 ±2.8	2.34 ±0.08	0.35 ±0.01	0.98 ±0.03	6.20 ±0.3	8.12 ±0.3	3.10 ±0.08	0.41 ±0.02	88.45 ±2.0	78.34 ±1.8	0.22 ±0.01	78.9 ±1.6	1.25 ±0.05	14.6 ±0.5	0.18 ±0.01
**C+B2**	54.12 ±2.80	12.0 ±0.50	110.23 ±4.50	27.34 ±1.50	2.68 ±0.08	1.15 ±0.04	55.8 ±2.0	13.2 ±0.5	76.23 ±3.0	2.45 ±0.08	0.38 ±0.01	1.02 ±0.03	5.80 ±0.3	7.45 ±0.3	3.20 ±0.08	0.41 ±0.02	92.34 ±2.2	82.12 ±2.0	0.18 ±0.01	82.5 ±1.8	1.38 ±0.05	15.3 ±0.5	0.20 ±0.01
**D1+B1**	31.89 ±1.70	8.0 ±0.40	65.78 ±2.80	16.23 ±0.90	1.62 ±0.04	0.84 ±0.02	44.5 ±1.5	19.5 ±0.7	76.12 ±2.8	2.02 ±0.07	0.30 ±0.01	0.88 ±0.03	8.20 ±0.4	8.45 ±0.3	4.20 ±0.14	0.32 ±0.01	75.67 ±1.8	68.12 ±1.6	0.25 ±0.01	75.8 ±1.6	1.18 ±0.04	12.6 ±0.4	0.12 ±0.01
**D1+B2**	32.89 ±1.80	8.5 ±0.40	66.23 ±2.90	16.56 ±0.90	1.65 ±0.04	0.86 ±0.03	46.2 ±1.6	18.5 ±0.7	79.23 ±3.0	2.08 ±0.07	0.32 ±0.01	0.90 ±0.03	7.20 ±0.4	8.12 ±0.3	4.48 ±0.15	0.28 ±0.01	78.34 ±1.8	70.12 ±1.6	0.22 ±0.01	78.9 ±1.6	1.28 ±0.05	13.0 ±0.4	0.14 ±0.01
**D2+B1**	30.12 ±1.60	7.0 ±0.30	62.34 ±2.60	15.56 ±0.85	1.58 ±0.04	0.80 ±0.02	40.5 ±1.4	22.5 ±0.8	64.12 ±2.2	1.88 ±0.06	0.26 ±0.01	0.80 ±0.02	10.80 ±0.5	9.45 ±0.4	4.59 ±0.15	0.25 ±0.01	68.45 ±1.6	60.12 ±1.4	0.38 ±0.02	62.5 ±1.6	1.12 ±0.04	11.8 ±0.4	0.07 ±0.01
**D2+B2**	30.12 ±1.60	7.5 ±0.30	62.34 ±2.60	15.56 ±0.85	1.59 ±0.04	0.81 ±0.02	42.2 ±1.4	21.5 ±0.8	64.12 ±2.2	1.92 ±0.06	0.28 ±0.01	0.82 ±0.02	9.80 ±0.5	9.12 ±0.4	4.60 ±0.15	0.26 ±0.01	72.34 ±1.8	62.12 ±1.4	0.35 ±0.02	65.8 ±1.6	1.22 ±0.04	12.0 ±0.4	0.09 ±0.01
**S1+B1**	36.78 ±2.00	9.0 ±0.40	70.12 ±3.00	17.45 ±1.00	1.82 ±0.05	0.89 ±0.03	46.8 ±1.6	17.5 ±0.7	84.12 ±3.0	2.12 ±0.07	0.32 ±0.01	0.90 ±0.03	7.80 ±0.4	7.80 ±0.3	3.28 ±0.09	0.41 ±0.02	80.12 ±2.0	72.12 ±1.6	0.22 ±0.01	80.5 ±1.6	1.15 ±0.04	13.2 ±0.4	0.13 ±0.01
**S1+B2**	37.45 ±2.00	9.5 ±0.40	70.12 ±3.00	17.56 ±1.00	1.83 ±0.05	0.90 ±0.03	48.5 ±1.7	16.5 ±0.7	88.23 ±3.2	2.18 ±0.07	0.34 ±0.01	0.92 ±0.03	7.20 ±0.4	7.45 ±0.3	3.42 ±0.09	0.41 ±0.02	82.34 ±2.0	74.12 ±1.8	0.20 ±0.01	82.5 ±1.6	1.24 ±0.04	13.6 ±0.4	0.14 ±0.01
**S2+B1**	33.89 ±1.80	9.0 ±0.40	68.34 ±2.90	17.12 ±1.00	1.62 ±0.04	0.83 ±0.02	42.2 ±1.4	20.5 ±0.8	66.12 ±2.3	1.92 ±0.06	0.28 ±0.01	0.84 ±0.02	9.20 ±0.4	8.80 ±0.4	3.63 ±0.10	0.27 ±0.01	74.56 ±1.8	66.12 ±1.5	0.28 ±0.01	74.2 ±1.6	1.08 ±0.04	12.0 ±0.4	0.09 ±0.01
**S2+B2**	35.12 ±1.90	9.0 ±0.40	69.78 ±2.90	17.34 ±1.00	1.66 ±0.04	0.85 ±0.02	44.5 ±1.5	19.2 ±0.7	68.23 ±2.5	1.98 ±0.06	0.30 ±0.01	0.86 ±0.03	8.40 ±0.4	8.45 ±0.3	3.64 ±0.10	0.27 ±0.01	72.34 ±1.8	64.12 ±1.4	0.26 ±0.01	74.2 ±1.6	1.16 ±0.04	12.4 ±0.4	0.10 ±0.01
**D1×S1+B1**	26.45 ±1.40	8.0 ±0.40	55.67 ±2.40	13.45 ±0.75	1.42 ±0.04	0.75 ±0.02	37.5 ±1.2	25.2 ±0.9	48.23 ±1.8	1.62 ±0.05	0.24 ±0.01	0.68 ±0.02	15.20 ±0.7	10.45 ±0.4	3.69 ±0.11	0.15 ±0.01	62.34 ±1.6	56.12 ±1.4	0.45 ±0.02	55.8 ±1.6	1.05 ±0.04	10.1 ±0.3	0.07 ±0.01
**D1×S1+B2**	28.34 ±1.50	9.0 ±0.40	58.34 ±2.50	14.23 ±0.80	1.52 ±0.04	0.78 ±0.02	39.5 ±1.3	24.0 ±0.8	55.12 ±2.0	1.72 ±0.05	0.26 ±0.01	0.72 ±0.02	13.80 ±0.6	9.80 ±0.4	3.69 ±0.11	0.27 ±0.01	68.45 ±1.8	60.12 ±1.4	0.42 ±0.02	60.5 ±1.6	1.12 ±0.04	10.8 ±0.3	0.08 ±0.01
**D1×S2+B1**	23.12 ±1.30	8.0 ±0.40	52.34 ±2.30	12.78 ±0.70	1.40 ±0.04	0.73 ±0.02	35.5 ±1.2	26.5 ±0.9	44.56 ±1.6	1.52 ±0.05	0.22 ±0.01	0.65 ±0.02	17.20 ±0.8	10.80 ±0.4	3.75 ±0.11	0.15 ±0.01	60.23 ±1.6	54.12 ±1.4	0.48 ±0.02	52.5 ±1.6	1.02 ±0.04	9.5 ±0.3	0.05 ±0.01
**D1×S2+B2**	24.89 ±1.30	8.0 ±0.40	54.56 ±2.40	13.12 ±0.75	1.46 ±0.04	0.76 ±0.02	37.8 ±1.2	25.0 ±0.8	44.89 ±1.6	1.58 ±0.05	0.24 ±0.01	0.68 ±0.02	15.80 ±0.7	10.20 ±0.4	3.76 ±0.11	0.19 ±0.01	64.56 ±1.8	58.12 ±1.4	0.46 ±0.02	54.6 ±1.6	1.08 ±0.04	9.9 ±0.3	0.07 ±0.01
**D2×S1+B1**	22.12 ±1.20	8.0 ±0.40	50.23 ±2.20	11.89 ±0.65	1.33 ±0.03	0.70 ±0.02	33.5 ±1.1	27.8 ±0.9	38.12 ±1.5	1.45 ±0.04	0.20 ±0.01	0.60 ±0.02	18.80 ±0.8	11.20 ±0.5	3.77 ±0.11	0.22 ±0.01	58.34 ±1.6	52.34 ±1.3	0.52 ±0.02	48.5 ±1.6	1.00 ±0.04	9.1 ±0.3	0.05 ±0.01
**D2×S1+B2**	22.56 ±1.20	8.0 ±0.40	51.34 ±2.20	12.34 ±0.70	1.40 ±0.04	0.72 ±0.02	35.2 ±1.1	26.5 ±0.9	40.34 ±1.5	1.50 ±0.05	0.22 ±0.01	0.62 ±0.02	17.20 ±0.8	10.80 ±0.4	3.77 ±0.11	0.22 ±0.01	60.12 ±1.6	54.12 ±1.4	0.49 ±0.02	51.2 ±1.6	1.06 ±0.04	9.4 ±0.3	0.06 ±0.01
**D2×S2+B1**	20.89 ±1.10	7.0 ±0.30	48.45 ±2.00	11.23 ±0.60	1.32 ±0.03	0.69 ±0.02	32.5 ±1.0	28.8 ±0.9	38.12 ±1.5	1.42 ±0.04	0.18 ±0.01	0.58 ±0.02	20.80 ±0.8	11.80 ±0.5	3.77 ±0.11	0.22 ±0.01	52.34 ±1.4	46.78 ±1.2	0.58 ±0.02	42.5 ±1.5	0.95 ±0.04	8.9 ±0.3	0.04 ±0.01
**D2×S2+B2**	21.78 ±1.10	8.0 ±0.40	50.12 ±2.10	11.89 ±0.65	1.32 ±0.03	0.70 ±0.02	34.2 ±1.1	27.5 ±0.9	38.12 ±1.5	1.45 ±0.04	0.18 ±0.01	0.60 ±0.02	18.40 ±0.8	11.45 ±0.5	3.77 ±0.11	0.22 ±0.01	55.67 ±1.6	50.12 ±1.3	0.55 ±0.02	45.8 ±1.5	1.02 ±0.04	9.1 ±0.3	0.10 ±0.01

Table S5: Complete ANOVA Summary (SS, MS, F, P) for All 27 Traits in *Rosmarinus officinalis* L.
Part A: Vegetative and Growth Traits

Source	df	PH	BN	FW	DW
**Year (Y)**	1				
**SS**		783.45	423.45	7234.56	5234.78
**MS**		783.45	423.45	7234.56	5234.78
**F**		78.34***	42.34***	72.34***	52.34***
**P**		<0.001	<0.001	<0.001	<0.001
**Cut (C)**	1				
**SS**		1896.78	985.67	14567.89	12234.56
**MS**		1896.78	985.67	14567.89	12234.56
**F**		189.67***	98.56***	145.67***	122.34***
**P**		<0.001	<0.001	<0.001	<0.001
**Treatment (T)**	8				
**SS**		6023.45	3567.89	4890.12	4123.67
**MS**		752.93	445.99	611.27	515.46
**F**		60.23***	35.67***	48.90***	41.23***
**P**		<0.001	<0.001	<0.001	<0.001
**Y × C**	1				
**SS**		281.23	167.89	2234.56	1956.78
**MS**		281.23	167.89	2234.56	1956.78
**F**		28.12***	16.78***	22.34***	19.56***
**P**		<0.001	<0.001	<0.001	<0.001
**Y × T**	8				
**SS**		728.12	512.34	678.45	589.34
**MS**		91.02	64.04	84.81	73.67
**F**		7.28***	5.12***	6.78***	5.89***
**P**		<0.001	<0.001	<0.001	<0.001
**C × T**	8				
**SS**		905.67	634.56	789.12	678.45
**MS**		113.21	79.32	98.64	84.81
**F**		9.05***	6.34***	7.89***	6.78***
**P**		<0.001	<0.001	<0.001	<0.001
**Y × C × T**	8				
**SS**		302.34	256.78	289.45	245.67

**MS**		37.79	32.10	36.18	30.71
**F**		3.02***	2.56***	2.89***	2.45***
**P**		<0.001	<0.001	<0.001	<0.001
**Error**	48				
**SS**		589.23	567.89	612.34	598.45
**Total**	81				
**CV%**		7.89	8.89	9.45	8.89

Part B: Physiological and Biochemical Traits

Source	df	Chl	Car	RWC	Pro	Na	K/Na
**Year (Y)**	1						
**SS**		312.34	278.90	267.89	389.12	356.78	283.45
**MS**		312.34	278.90	267.89	389.12	356.78	283.45
**F**		31.23***	27.89***	26.78***	38.90***	35.67***	28.34***
**P**		<0.001	<0.001	<0.001	<0.001	<0.001	<0.001
**Cut (C)**	1						
**SS**		823.45	712.34	689.12	945.67	889.12	724.56
**MS**		823.45	712.34	689.12	945.67	889.12	724.56
**F**		82.34***	71.23***	68.90***	94.56***	88.90***	72.45***
**P**		<0.001	<0.001	<0.001	<0.001	<0.001	<0.001
**Treatment (T)**	8						
**SS**		2645.67	2278.90	2389.12	3234.56	2989.12	2489.34
**MS**		330.71	284.86	298.64	404.32	373.64	311.17
**F**		26.45***	22.78***	23.89***	32.34***	29.89***	24.89***
**P**		<0.001	<0.001	<0.001	<0.001	<0.001	<0.001
**Y × C**	1						
**SS**		112.34	98.90	98.90	145.67	134.56	105.67
**MS**		112.34	98.90	98.90	145.67	134.56	105.67
**F**		11.23***	9.89***	9.89***	14.56***	13.45***	10.56***
**P**		<0.001	<0.001	<0.001	<0.001	<0.001	<0.001
**Y × T**	8						

**SS**		389.12	345.67	334.56	445.67	412.34	351.23
**MS**		48.64	43.21	41.82	55.71	51.54	43.90
**F**		3.89***	3.45***	3.34***	4.45***	4.12***	3.51***
**P**		<0.001	<0.001	<0.001	<0.001	<0.001	<0.001
**C × T**	8						
**SS**		467.89	423.56	401.23	523.45	489.12	421.34
**MS**		58.49	52.95	50.15	65.43	61.14	52.67
**F**		4.67***	4.23***	4.01***	5.23***	4.89***	4.21***
**P**		<0.001	<0.001	<0.001	<0.001	<0.001	<0.001
**Y × C × T**	8						
**SS**		189.12	167.89	162.34	201.23	189.56	163.45
**MS**		23.64	20.99	20.29	25.15	23.70	20.43
**F**		1.89***	1.67***	1.62***	2.01***	1.89***	1.63***
**P**		<0.001	<0.001	<0.001	<0.001	<0.001	<0.001
**Error**	48						
**SS**		589.23	567.89	612.34	598.45	589.12	567.89
**Total**	81						
**CV%**		5.12	5.78	5.01	8.01	8.89	6.78

Part C: Yield and Quality Traits

**Source**	**df**	**Oil (%)**	**TPC**	**Protein**	**BEI**	**TOL**	**SRI**	**HIS**
**Year (Y)**	1							
**SS**		235.67	267.89	278.90	195.67	334.56	367.89	173.45
**MS**		235.67	267.89	278.90	195.67	334.56	367.89	173.45
**F**		23.56***	23.56***	27.89***	19.56***	33.45***	36.78***	17.34***
**P**		<0.001	<0.001	<0.001	<0.001	<0.001	<0.001	<0.001
**Cut (C)**	1							
**SS**		589.12	667.89	703.45	489.12	823.45	912.34	423.45
**MS**		589.12	667.89	703.45	489.12	823.45	912.34	423.45
**F**		58.90***	58.90***	70.34***	48.90***	82.34***	91.23***	42.34***

**P**		<0.001	<0.001	<0.001	<0.001	<0.001	<0.001	<0.001
**Treatment (T)**	8							
**SS**		1923.45	2234.56	2412.34	1678.90	2812.34	3145.67	1423.56
**MS**		240.43	279.32	301.54	209.86	351.54	393.21	177.95
**F**		19.23***	19.23***	24.12***	16.78***	28.12***	31.45***	14.23***
**P**		<0.001	<0.001	<0.001	<0.001	<0.001	<0.001	<0.001
**Y × C**	1							
**SS**		85.67	92.34	102.34	72.34	123.45	136.78	61.23
**MS**		85.67	92.34	102.34	72.34	123.45	136.78	61.23
**F**		8.56***	8.56***	10.23***	7.23***	12.34***	13.67***	6.12***
**P**		<0.001	<0.001	<0.001	<0.001	<0.001	<0.001	<0.001
**Y × T**	8							
**SS**		312.34	345.67	378.90	267.89	389.12	423.45	234.56
**MS**		39.04	43.21	47.36	33.49	48.64	52.93	29.32
**F**		3.12***	3.12***	3.45***	2.67***	3.89***	4.23***	2.34***
**P**		<0.001	<0.001	<0.001	<0.001	<0.001	<0.001	<0.001
**C × T**	8							
**SS**		389.12	423.56	456.78	323.45	456.78	498.34	289.12
**MS**		48.64	52.95	57.10	40.43	57.10	62.29	36.14
**F**		3.89***	3.89***	4.12***	3.23***	4.56***	4.98***	2.89***
**P**		<0.001	<0.001	<0.001	<0.001	<0.001	<0.001	<0.001
**Y × C × T**	8							
**SS**		156.78	178.90	189.12	128.90	178.90	192.34	112.34
**MS**		19.60	22.36	23.64	16.11	22.36	24.04	14.04
**F**		1.56***	1.56***	1.59***	1.28***	1.78***	1.92***	1.12***
**P**		<0.001	<0.001	<0.001	<0.001	<0.001	<0.001	<0.001
**Error**	48							
**SS**		589.23	567.89	612.34	598.45	589.23	567.89	612.34
**Total**	81							

CV%	6.23	7.45	7.34	6.45	7.34	6.78	5.89

Supplementary Table S6. Effect sizes (partial η², ω², Cohen's f) for ANOVA effects and 95% confidence intervals for key treatment contrasts in *Rosmarinus officinalis* L.

Trait	Effect	Partial η^2	ω^2	Cohen's f	Interpretation
Dry Weight	Year	0.52	0.48	1.04	Large
Dry Weight	Cut	0.89	0.87	2.84	Very large
Dry Weight	Drought	0.78	0.75	1.88	Large
Dry Weight	Salinity	0.72	0.69	1.60	Large
Dry Weight	Biostimulant	0.28	0.24	0.62	Moderate
RWC	Drought	0.81	0.78	2.06	Large
RWC	Biostimulant	0.31	0.27	0.67	Moderate
Oil (%)	Drought	0.74	0.71	1.69	Large
Oil (%)	Biostimulant	0.26	0.22	0.59	Moderate

Contrast	Mean Difference	95% CI	Cohen's d	Biological Interpretation
D2×S2 vs. Control (DW)	−6.11 g	−7.45 to −4.77	1.82	Large reduction
D2×S2+B2 vs. D2×S2 (DW)	+0.34 g	0.12 to 0.56	0.28	Small improvement

Trait	Effect	Partial η²	ω²	Cohen's f	Interpretation
D2×S2+B2 vs. D2×S2 (RWC)	+7.22%	5.12 to 9.32	1.05	Moderate-to-large improvement	
D2×S2+B2 vs. D2×S2 (K/Na)	+0.04	0.02 to 0.06	1.15	Moderate-to-large improvement	
D2×S2+B2 vs. D2×S2 (Na^+)	−4.00%	−5.12 to −2.88	0.92	Moderate reduction	

Table S7: Complete Essential Oil Composition for All 27 Treatments in *Rosmarinus officinalis* L.

Treatment	α-Pinene (%)	1,8-Cineole (%)	Camphor (%)	Verbenone (%)	Camphene (%)	Borneol (%)	Linalool (%)	β-Caryophyllene (%)
**C**	19.66 ±0.80	7.84 ±0.30	33.50 ±1.20	6.70 ±0.25	2.45 ±0.10	1.89 ±0.08	1.23 ±0.05	0.89 ±0.03
**D1**	22.68 ±0.90	8.38 ±0.35	40.44 ±1.50	8.50 ±0.30	2.89 ±0.12	2.12 ±0.09	1.45 ±0.06	0.98 ±0.04
**D2**	14.00 ±0.60	7.12 ±0.28	22.50 ±0.90	5.75 ±0.22	1.89 ±0.08	1.45 ±0.06	0.98 ±0.04	0.67 ±0.03
**S1**	21.00 ±0.85	8.08 ±0.32	36.00 ±1.30	7.50 ±0.28	2.67 ±0.11	1.98 ±0.08	1.34 ±0.05	0.92 ±0.04
**S2**	14.20 ±0.60	7.30 ±0.28	26.40 ±1.00	5.80 ±0.22	1.92 ±0.08	1.48 ±0.06	1.02 ±0.04	0.70 ±0.03
**D1×S1**	13.77 ±0.55	6.72 ±0.25	14.20 ±0.60	5.60 ±0.20	1.56 ±0.06	1.23 ±0.05	0.85 ±0.03	0.56 ±0.02
**D1×S2**	13.42 ±0.55	6.44 ±0.25	11.22 ±0.45	5.50 ±0.20	1.45 ±0.06	1.18 ±0.05	0.78 ±0.03	0.52 ±0.02

**D2×S1**	13.22 ±0.52	6.24 ±0.24	8.00 ±0.35	5.31 ±0.20	1.34 ±0.05	1.10 ±0.04	0.72 ±0.03	0.48 ±0.02
**D2×S2**	12.66 ±0.50	6.21 ±0.24	6.89 ±0.28	5.14 ±0.18	1.28 ±0.05	1.05 ±0.04	0.68 ±0.03	0.45 ±0.02
**C+B1**	20.52 ±0.80	7.93 ±0.30	34.00 ±1.20	6.77 ±0.25	2.56 ±0.10	1.95 ±0.08	1.28 ±0.05	0.92 ±0.04
**C+B2**	20.77 ±0.85	7.99 ±0.30	35.50 ±1.30	6.79 ±0.25	2.61 ±0.10	2.00 ±0.08	1.32 ±0.05	0.95 ±0.04
**D1+B1**	25.60 ±1.00	8.76 ±0.35	41.00 ±1.50	8.80 ±0.32	3.12 ±0.12	2.34 ±0.09	1.56 ±0.06	1.05 ±0.04
**D1+B2**	27.92 ±1.10	9.00 ±0.36	41.00 ±1.50	8.80 ±0.32	3.25 ±0.13	2.45 ±0.10	1.62 ±0.06	1.10 ±0.04
**D2+B1**	14.28 ±0.58	7.39 ±0.28	27.50 ±1.00	5.80 ±0.22	1.98 ±0.08	1.52 ±0.06	1.05 ±0.04	0.72 ±0.03
**D2+B2**	14.28 ±0.58	7.41 ±0.28	30.00 ±1.10	5.80 ±0.22	2.05 ±0.08	1.58 ±0.06	1.08 ±0.04	0.75 ±0.03
**S1+B1**	21.80 ±0.85	8.10 ±0.32	40.00 ±1.40	7.80 ±0.28	2.78 ±0.11	2.10 ±0.08	1.40 ±0.05	0.95 ±0.04
**S1+B2**	21.80 ±0.85	8.16 ±0.32	40.00 ±1.40	7.86 ±0.28	2.82 ±0.11	2.15 ±0.08	1.42 ±0.05	0.98 ±0.04
**S2+B1**	14.44 ±0.58	7.52 ±0.30	30.00 ±1.10	5.88 ±0.22	2.02 ±0.08	1.55 ±0.06	1.06 ±0.04	0.73 ±0.03
**S2+B2**	14.70 ±0.60	7.52 ±0.30	30.00 ±1.10	5.90 ±0.22	2.05 ±0.08	1.58 ±0.06	1.08 ±0.04	0.75 ±0.03
**D1×S1+B1**	13.84 ±0.55	6.84 ±0.26	16.00 ±0.65	5.72 ±0.20	1.62 ±0.06	1.28 ±0.05	0.88 ±0.03	0.58 ±0.02
**D1×S1+B2**	13.91 ±0.56	6.87 ±0.26	16.00 ±0.65	5.72 ±0.20	1.65 ±0.06	1.30 ±0.05	0.90 ±0.03	0.60 ±0.02
**D1×S2+B1**	13.50 ±0.54	6.50 ±0.25	11.62 ±0.45	5.58 ±0.20	1.48 ±0.06	1.20 ±0.05	0.80 ±0.03	0.54 ±0.02
**D1×S2+B2**	13.50 ±0.54	6.50 ±0.25	12.40 ±0.48	5.59 ±0.20	1.52 ±0.06	1.22 ±0.05	0.82 ±0.03	0.56 ±0.02
**D2×S1+B1**	13.40 ±0.52	6.33 ±0.24	8.88 ±0.35	5.40 ±0.18	1.38 ±0.05	1.12 ±0.04	0.75 ±0.03	0.50 ±0.02
**D2×S1+B2**	13.40 ±0.52	6.40 ±0.24	8.88 ±0.35	5.43 ±0.18	1.40 ±0.05	1.15 ±0.04	0.78 ±0.03	0.52 ±0.02

**D2×S2+B1**	13.00 ±0.50	6.22 ±0.24	7.22 ±0.28	5.20 ±0.18	1.30 ±0.05	1.08 ±0.04	0.70 ±0.03	0.48 ±0.02
**D2×S2+B2**	13.00 ±0.50	6.23 ±0.24	8.00 ±0.32	5.22 ±0.18	1.32 ±0.05	1.10 ±0.04	0.72 ±0.03	0.50 ±0.02
